# The *Aeromonas salmonicida* subsp. *salmonicida* exoproteome: global analysis, moonlighting proteins and putative antigens for vaccination against furunculosis

**DOI:** 10.1186/1477-5956-11-44

**Published:** 2013-10-15

**Authors:** Philippe Vanden Bergh, Manfred Heller, Sophie Braga-Lagache, Joachim Frey

**Affiliations:** 1 Institute of Veterinary Bacteriology, University of Bern, Länggassstrasse 122, P.O. Box 8466, 3001 Bern, Switzerland; 2 Department of Clinical Research, University of Bern, P.O. Box 37, 3010 Bern, Switzerland

## Abstract

**Background:**

*Aeromonas salmonicida* subsp. *salmonicida*, the etiologic agent of furunculosis, is a major pathogen of fisheries worldwide. Despite the identification of several virulence factors the pathogenesis is still poorly understood. We have used high-throughput proteomics to display the differences between in vitro secretome of *A. salmonicida* wild-type (wt, hypervirulent, JF5054) and T3SS-deficient (isogenic Δ*ascV*, extremely low-virulent, JF2747) strains in exponential (GP) and stationary (SP) phases of growth.

**Results:**

Among the different experimental conditions we obtained semi-quantitative values for a total of 2136 *A. salmonicida* proteins. Proteins of specific *A. salmonicida* species were proportionally less detected than proteins common to the *Aeromonas* genus or those shared with other *Aeromonas* species, suggesting that in vitro growth did not induce the expression of these genes. Four detected proteins which are unidentified in the genome of reference strains of *A. salmonicida* were homologous to components of the conjugative T4SS of *A. hydrophila* pRA1 plasmid. Polypeptides of three proteins which are specific to the 01-B526 strain were also discovered. In supernatants (SNs), the number of detected proteins was higher in SP (326 for wt vs 329 for mutant) than in GP (275 for wt vs 263 for mutant). In pellets, the number of identified proteins (a total of 1536) was approximately the same between GP and SP. Numerous highly conserved cytoplasmic proteins were present in *A. salmonicida* SNs (mainly EF-Tu, EF-G, EF-P, EF-Ts, TypA, AlaS, ribosomal proteins, HtpG, DnaK, peptidyl-prolyl cis-trans isomerases, GAPDH, Enolase, FbaA, TpiA, Pgk, TktA, AckA, AcnB, Mdh, AhpC, Tpx, SodB and PNPase), and several evidences support the theory that their extracellular localization was not the result of cell lysis. According to the Cluster of Orthologous Groups classification, 29% of excreted proteins in *A. salmonicida* SNs were currently poorly characterized.

**Conclusions:**

In this part of our work we elucidated the whole *in vitro* exoproteome of hypervirulent *A. salmonicida* subsp. *salmonicida* and showed the secretion of several highly conserved cytoplasmic proteins with putative moonlighting functions and roles in virulence. All together, our results offer new information about the pathogenesis of furunculosis and point out potential candidates for vaccine development.

## Background

*Aeromonas salmonicida* subsp. *salmonicida*, a Gram-negative bacterium, is the etiologic agent of furunculosis, a frequent and major pathogen of fisheries worldwide which is generating significant economic losses related to deficits in zootechnical profits and the intensive use of antibiotics [1]. *A. salmonicida* causes severe septicaemia and acute mortality in susceptible salmonid hosts [2]. The sub-acute or chronic form of the disease is characterized by the presence of lesions resembling boils, i.e. furuncles, in the musculature [2]. Despite the publication of the genome of the *A. salmonicida* A449 reference strain in 2008 [3] and the identification of several virulence factors, the pathogenesis is still poorly understood and needs further investigation. The type three secretion system (T3SS) of *A. salmonicida* is recognized as having a major effect on virulence, as independent studies have shown that isogenic mutant strains for T3SS structural proteins are non-virulent both in vitro and in vivo [4-8]. However only four T3SS effectors have been identified, and they do not seem to be solely responsible for *Aeromonas* virulence because individual knock-out mutations of these genes [6] or a triple-effector knock-out mutant [9] keep a virulent phenotype. Additional virulence T3SS effectors should therefore be involved in the pathogenesis process.

Proteomics has been successfully used to study the impact on the bacterial extracellular proteome (secretome) of targeted gene deletion in secretion systems [10]. In the same way, the aim of this work was to use high-throughput proteomics to display the secretome of *A. salmonicida* subsp. *salmonicida* wild-type (wt, hypervirulent) and an isogenic T3SS-deficient mutant (*ΔascV*, extremely low-virulent) during the exponential-growth phase (GP) and stationary phase (SP). Results and discussions are separated into two parts. In this first part, the authors focus on the general analysis of data, the discovery of cytoplasmic proteins with putative moonlighting activities in supernatants of *A. salmonicida* and the identification of putative antigens for fish vaccination against furunculosis. In the second part, the authors characterize the whole in vitro repertoire of T3SS effectors and discuss the roles of the well-described and new putative virulence factors of *A. salmonicida*[11].

## Results and discussion

### Comprehensive analysis of the *A. salmonicida* secretome

**Figure 1:**
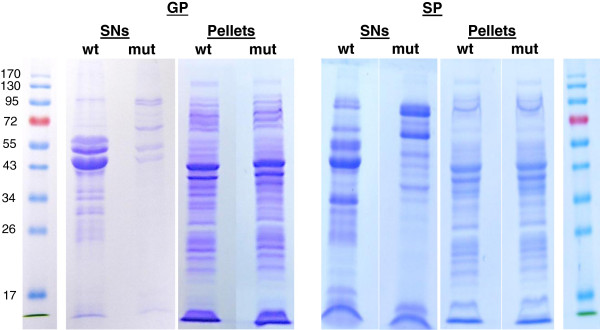
**SDS gel electrophoresis of *A. salmonicida *subsp. *salmonicida *proteins stained with Coomassie blue.** SDS gel electrophoresis of proteins from supernatants (SN) and pellets of wild-type (wt) and Δ*ascV* mutant (mut) strains in exponential (GP) and stationary phases (SP) of growth. The molecular weights (kDa) of the Protein Ladder are shown on both sides of the figure.

The extracellular proteins of exponential and stationary growth phase cultures of *A. salmonicida* were separated from bacterial pellets by centrifugation and concentrated from identical volumes of filtrated cell-free culture supernatant (SN) as described in Materials and Methods. Proteins derived from this concentration method and from bacterial pellets were separated by SDS-PAGE and revealed a clear difference in patterns between wt and *ΔascV* mutant SNs (Figure 1). Gels were then sliced and their protein contents were analysed by using high-throughput LC-MS/MS.

**Figure 2:**
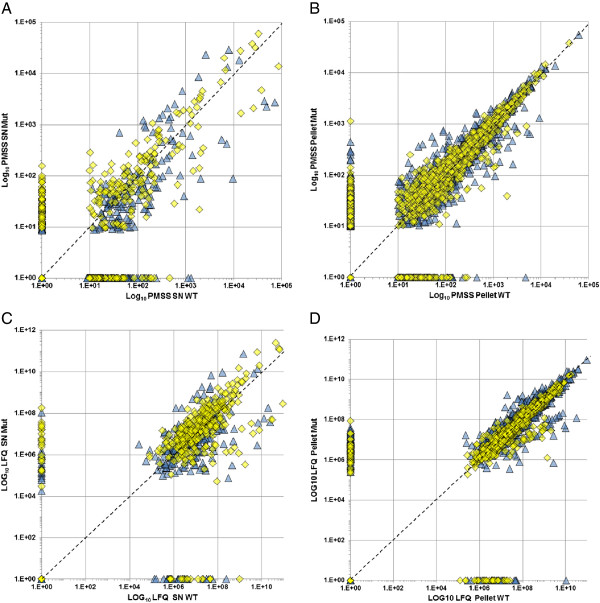
**Correlation of protein contents between wt and T3SS-mutant.** Each plot represents the PMSS **(A** and **B)** or LFQ **(C** and **D)** values for each protein identified in wt (X-axis) and/or mutant (Y-axis) strains, in supernatants **(**SNs, **A** and **C)** and pellets **(B** and **D)**. Values of exponential growth phase (GP) are dark blue triangles, and stationary phase (SP) values are yellow squares. The global distribution of wt vs mutant protein values was linear in all conditions, but with a larger repartition in SNs than in pellets, thereby indicating differences in protein secretion between wt and Δ*ascV* strains.

We identified a total of 2136 *A. salmonicida* proteins with PMSS and LFQ values among the different experimental conditions (see Methods for explanations and tables in Additional files 1, 2, 3, 4, 5 and 6 for raw data) for 1861 and 2070 proteins respectively (Figure 2 and Additional file 7). These values correspond to a semi-quantitative abundance estimate of protein species present in SDS-PAGE gels and were used as a surrogate for the amount of secreted proteins in TCA-concentrated SNs and the amount of produced proteins in whole pellets. The correlation of PMSS and LFQ values between wt vs *ΔascV* mutant strains was linear in any conditions, but with a larger repartition in SNs than in pellets (Figures 2A and C), thus indicating differences in protein secretion between the wt and *ΔascV* strains.

**Figure 3:**
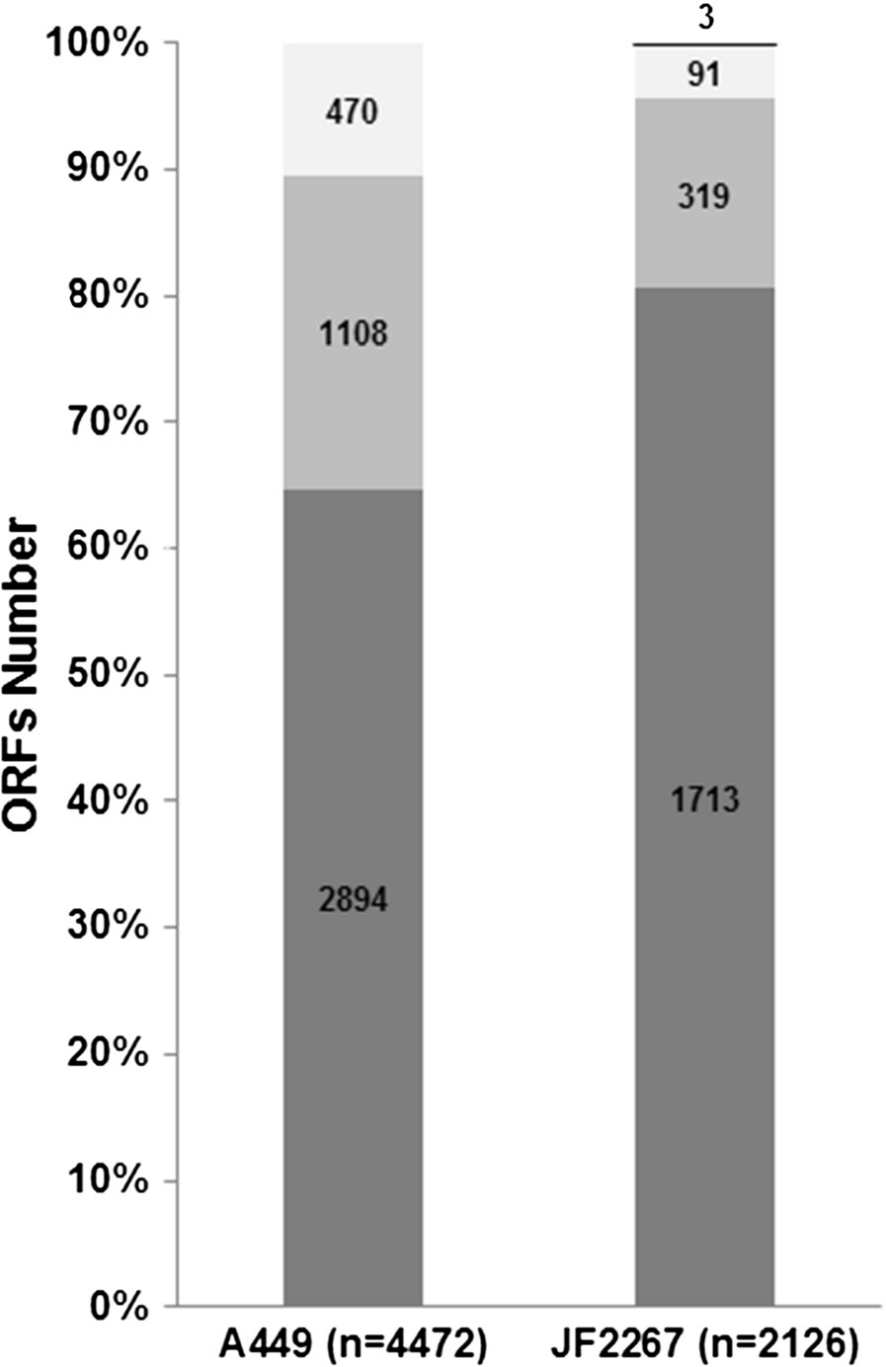
**Identified proteins common, shared or specific to *A. salmonicida.*** The diagram shows the proportion of JF2267 proteins identified by MS as common to the genus *Aeromonas* (dark grey), shared with other species (medium grey), specific to *Aeromonas salmonicida* subsp. *salmonicida* (light grey) or specific to JF2267 (black) in comparison to the reference strain A449.

Most of the identified proteins (81%) were common to the *Aeromonas* genus, while 15% were shared with at least one other *Aeromonas* species and 4% were specific to *A. salmonicida* (Figure 3). In comparison, the theoretical genome of *A. salmonicida* A449 [GenBank: CP000644.1, CP000645.1, CP000646.1, AY301063.1, AY301064.1 and AY301065.1] [3] predicts that common, shared and specific proteins represent 65%, 25% and 10% of the total proteome respectively, while our results showed that 59%, 29% and 19% of *A. salmonicida* A449 common, shared and specific proteins were identified in our experiment. We hypothesize that the cultivation of bacteria in the TSB medium without specific restrictions and without the presence of host cells might explain the lower identification of specific proteins necessary for the adaptation of peculiar niches, and that these would mainly reside among genes shared with other *Aeromonas* species or specific to *A. salmonicida*.

The MS analysis also found polypeptides for four proteins which are homologous to components of the type four conjugative transfer system (T4SS) of *A. hydrophila* pRA1 plasmid (pRA1_0073, pRA1_0074, pRA1_0075/TraC and pRA1_0077/TrhF), suggesting that a IncA/C plasmid variant might be present in *A. salmonicida* JF2267. Such plasmids are able to confer multidrug-resistance to *A. salmonicida*[12]. Fragments of three proteins specific of *A. salmonicida* subsp. *salmonicida* 01-B526 (IYQ_00957/RdgC, *A. veronii* AMC35 HMPREF1170_02622 and *Vibrio cholerae* 1587 A55_1878 homologues) which are predicted to be encoded in the contig two [GenBank: AGVO01000002.1] were also discovered.

**Figure 4:**
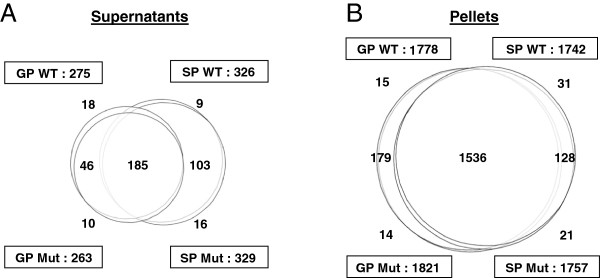
**Venn diagrams summarizing proteins identified for each condition.** Venn diagrams were produced from identified proteins with LFQ values in supernatants **(**SNs, **A)** and pellets **(B)**, in either exponential phase of growth (GP) or stationary phase (SP) for wild-type (wt) and mutant (mut) strains. The number of proteins detected in SNs and pellets of wt and T3SS mutant strains was similar, but the most important differences in protein content were observed between GP and SP.

The majority of proteins detected in SNs and pellets of wt and T3SS mutant strains were similar, but the most important differences in protein content were observed between GP and SP (Figure 4, for LFQ values). In SNs, the number of detected proteins was higher in SP (326 for wt vs 329 for mutant) than in GP (275 for wt vs 263 for mutant). A total of 185 proteins were identified in SNs of all conditions (wt or mut, GP or SP), and 46 were specific to the GP SNs and 103 specific to the SP SNs. In pellets, the number of identified proteins was approximately the same between GP and SP, with a total of 1536 proteins identified in pellets of all conditions (wt or mut, GP or SP), of which 179 were specific to the GP pellets and 128 were specific to the SP pellets.

### Subcellular localization and COG classification of detected *A. salmonicida* proteins

**Figure 5:**
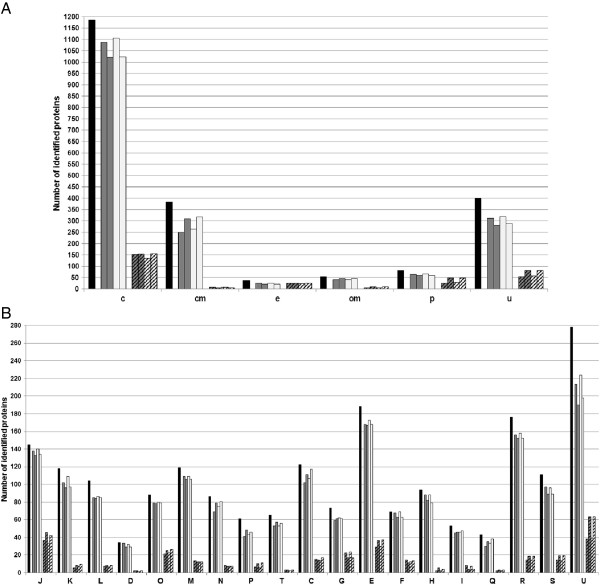
**Subcellular localization and COG classification of identified proteins.** Identified proteins were classified according to their subcellular localization **(A)** and Cluster of Orthologous Group **(B)**. In each diagram the first dark column represents the total number of proteins identified in the group. Grey and white columns are wt and mutant values of pellets in either GP (first column) or SP (second column) respectively. Hatched columns are proteins identified in SNs. In diagram **A**, c = cytoplasmic, cm = cytoplasmic membrane, e = extracellular, om = outer membrane, p = periplasmic and u = unknown localization. In diagram **B**, J = translation, ribosomal structure and biogenesis, K = transcription, L = DNA replication, recombination and repair, D = cell division and chromosome partitioning, O = posttranslational modification, protein turnover, chaperones, M = cell envelope biogenesis, outer membrane, N = cell motility and secretion, P = inorganic ion transport and metabolism, T = signal transduction mechanisms, C = energy production and conversion, G = carbohydrate transport and metabolism, E = amino acid transport and metabolism, F = nucleotide transport and metabolism, H = coenzyme metabolisme, I = lipid metabolism, Q = secondary metabolites biosynthesis, transport and catabolism, R = poorly characterized with general function prediction, S = poorly characterized with unknown function, and U = unclassified group.

**Figure 6:**
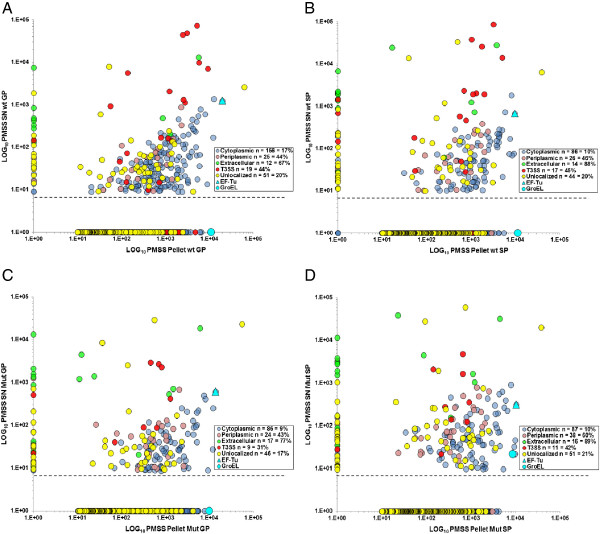
**Distribution of quantity values for each protein identified by MS according to their subcellular localization.** Each plot represents PMSS values for each protein identified in pellets (X-axis) and/or supernatants (SNs, Y-axis) for wt **(A** and **B)** and mutant **(C** and **D)** strains, in either the exponential phase of growth **(**GP, **A** and **C)** or the stationary phase **(**SP, **B** and **D)**. Numerous cytoplasmic and periplasmic proteins were present in SNs. EF-Tu and GroEL were among the most important proteins in all pellets, but they did not show the same levels in SNs. While GroEL (a marker of cell lysis) was never present in SNs **(**or in a very low quantity in **D)**, EF-Tu was abundantly identified in the SN of all conditions.

To predict the subcellular localization of 2136 detected proteins we employed the pSORTb v3.0.2 server [13] allocating the proteins in six groups (Figure 5A). Almost all of the identified proteins found by MS were detected at least once in pellets (wt or mut, GP or SP). The number of detected cytoplasmic, unlocalized, periplasmic and extracellular proteins was increased in GP pellets, while CM and OM proteins were increased in SP pellets. Among the proteins detected in SNs (wt or mut, GP or SP), the most represented were cytoplasmic (228/1185 = 19%), unlocalized (99/400 = 25%), periplasmic (54/80 = 67%) and extracellular proteins (28/36 = 78%) (Figure 5A). The number of detected unlocalized, periplasmic and OM proteins was increased in SP SNs (wt or mutant), whereas cytoplasmic proteins were only decreased in mutant GP SN. Predicted cytoplasmic proteins represented half of the detected components in SNs (228/442 = 52%) (Figure 5A). Our results showed that 83% (GP) and 90% (SP) of predicted cytoplasmic proteins identified by MS in pellets were never present in wt SNs (Figure 6), and we assume that the presence of predicted cytoplasmic proteins in SNs is not linked to cell lysis. To support this conclusion, we develop the example of well-characterized elongation factor Tu (EF-Tu or TufA) and GroEL chaperonin (HSP60) further. They were among the top seven most abundant *A. salmonicida* proteins present in similar amount in pellets, but GroEL was totally absent from SNs while EF-Tu was present in a high quantity in culture media (Figure 6). In accordance with our results, GroEL was localized to the bacterial cytoplasma and membranes in other bacteria [14,15] and was used as an indicator of cell lysis [16], while EF-Tu was frequently detected in bacterial SNs (Additional file 8). Moreover, the EF-Tu amount in our SNs (wt or mut) was lower in SP vs GP. The fact that EF-Tu did not accumulate in SNs also corroborates our conclusion that it was not a product of cell lysis. The same observation was made in the secretome of *Rhizobium etli* by Meneses and collaborators [17].

A partial list of conserved cytoplasmic proteins that were unexpectedly present in a high quantity in *A. salmonicida* SNs is given in the table of the Additional file 8. These elements belong to the translation (EF-Tu, elongation factor G [EF-G, FusA], EF-P, EF-Ts, TypA [or BipA] and alanyl tRNA synthetase [AlaS]), ribosomal structure (30S [S1, S5, S8 and S16] and 50S [L1, L3, L6 and L11]), chaperones (HtpG [HSP90], DnaK [HSP70], peptidyl-prolyl cis-trans isomerases [PpiA, -B and -C], and trigger factor [Tig]), pathways of glucose metabolism and citrate cycle (glyceraldehyde-3-phosphate dehydrogenase [Gap, GAPDH], enolase [Eno], fructose-biphosphate aldolase class II [FbaA], triose phosphate isomerase [TpiA], phosphoglycerate kinase [Pgk], transketolase A [TktA]), acetate kinase [AckA], aconitate hydratase B [AcnB], malate dehydrogenase [Mdh]), antioxidant enzyme systems (alkyl hydroperoxide reductase C [AhpC], a thiol peroxidase [Tpx], and superoxide dismutase B [SodB]), and a polyribonucleotide nucleotidyltransferase [PNPase]. The extracellular presence of most of these *A. salmonicida* proteins is confirmed by a precedent study [18] analysing the proteome associated to the cell surface (surfacome) of the bacterium (Additional file 9). In our MS analysis we detected 93% (66/71) of the proteins identified by Ebanks and collaborators [18] associated to the *A. salmonicida* OM, and 32% (23/71) were found in our SNs, thereby confirming that they are released in the medium. Interestingly, the majority of these highly conserved cytoplasmic proteins with atypical localization were already identified as homologues in the extracellular proteome of other bacteria (Additional file 8), and many were described as extracellular moonlighting components playing a role in bacterial virulence [19,20]. Moonlighting proteins constitute a subset of multifunctional proteins in which two or more functions cannot be ascribed to the fusion of genes encoding proteins with distinct functions, splice variants, or fragments of proteins that serve different functions after proteolysis [19]. For example, at least EF-Tu, DnaK, GAPDH, Eno, Pgk and Tpx were demonstrated to bind to plasminogen which have a complement-inhibitory activity, thereby providing an explanation for why pathogenic bacteria utilize components binding this protein for immune evasion [21,22]. Several of these proteins were also involved in binding to components of the host extracellular matrix (fibronectin, laminin, collagens) or the eukaryotic cell (membrane receptors, actin) playing a role in tissue adhesion and penetration, phagocytosis inhibition or immune evasion (Additional file 8). Ribosomal proteins have been found in the exoproteome of many bacteria, and mounting evidence points to their alternative extracellular location where they would perform nonribosomal functions [23]. The presence of aminoacyl tRNA synthetases in the bacterial exoproteome was also surprising, but some parasites secrete these components to modulate host inflammatory and immune responses [24-26]. Peptidyl-prolyl isomerases are FKBP domain-containing proteins which are reported as virulence factors in several pathogenic bacteria [27-29]. Some parasites also use these FKBP for virulence (Mip FKBP of *Trypanosoma cruzi*) and host immunomodulation (18-kDa cyclophilin of *Toxoplasma gondii*) [30]. Interestingly, all of these bacterial proteins have similar molecules in eukaryotes where they also exert moonlighting activities [19,20,31-36], and it might be possible that they are released in the extracellular medium in order to mimic and manipulate the functions of their eukaryotic homologues.

The mechanism explaining the secretion of these bacterial cytoplasmic proteins in the extracellular environment remains unclear. One hypothesis is that they might be secreted within outer membrane vesicles (OMVs) [17,37]. Pathogenic bacteria use these extracellular vesicles to manipulate host responses and, deliver virulence factors directly into eukaryotic cytosol [38-40]. Such nanovesicles were observed in *A. salmonicida* with an estimated size of 10 to 300 nm [41], suggesting that they might be present in the filtrated SNs. Their proteomic content is currently not known. Choi and collaborators identified 338 proteins associated to *Pseudomonas aeruginosa* OMVs [42] and EF-Tu, EF-G, ribosomal proteins (30S S1, S5 and 50S L1 and L11), HtpG, DnaK, Tig, AcnB and AhpC were present in these nanovesicles (Additional file 10). In fact, we found that 72% (242/338) of *P. aeruginosa* OMV proteins had homologues in *A. salmonicida* A449, and our MS analysis detected 86% (208/242) of these *P. aeruginosa* homologues but only 30% (62/208) were identified in our SNs (at least once in wt or mut, GP or SP). A large part of cytoplasmic (44), CM (53) and OM (28) proteins from *P. aeruginosa* OMVs were only detected in our pellets and would have to be detected in our SNs if *A. salmonicida* OMVs were present. Moreover, many *Pseudomonas* proteins that are abundant in OMVs have *A. salmonicida* homologues that were not detected in our SNs. As a result, we cannot conclude with certainty that unexpected proteins present in our SNs were associated to *A. salmonicida* OMVs, and this needs further studies before it can be confirmed.

The number of periplasmic and unlocalized components was also important and increased in SNs (wt or mut) (Figure 5A). Among periplasmic proteins, 50% (27/54) were associated to ABC transporters implied in the uptake of nutrients. The release of periplasmic proteins in the extracellular medium was also observed in other bacteria [43]. Only a few SN proteins were predicted to come from the CM (22/382 = 5%) or the OM (12/53 = 23%), and a detailed analysis of these components showed that they were mostly present in a very low amount or were hydrosoluble proteins with no transmembrane domain that would be misclassified.

We grouped 2093 identified proteins (excepted 43 components of the T3SS) according to the COG classification (Cluster of Orthologous Groups), and allocated them to 19 functional groups (Figure 5B). Larger groups of proteins found in SNs (425) were the “unclassified” (U = 79/291 identified = 27%), “translation, ribosomal structure and biogenesis” (J = 51/146 identified = 35%), “amino acid transport and metabolism” (E = 48/192 identified = 25%), “posttranslational modification, protein turnover, chaperones” (O = 30/87 identified = 34%), “carbohydrate transport and metabolism” (G = 26/73 identified = 36%), “energy production and conversion” (C = 23/123 identified = 19%), “unknow function” (S = 23/112 = 21%), and “general function prediction only” (R = 22/183 = 12%). Therefore the number of poorly characterized proteins (U, S and R groups) identified in *A. salmonicida* SNs (wt or mut, GP or SP) was relatively high (124), representing 29% of observed proteins. The proportion of these categories was also increased in SNs in comparison to pellets.

### Identification of putative protective antigens for fish immunization against furunculosis

Only a few proteins have currently been shown to confer a partial protection to fish when inoculated as a subunit vaccine. VapA induces a variable level of protective immunization (Additional file 11), but the maximum protection was obtained when fish were infected by *A. salmonicida* strains secreting homologous VapA proteins [44]. The S-layer protein therefore seems much too specific to be used in vaccine formulation against all *A. salmonicida* species. Vaccination with OmpAI also gave a partial protection, but was inferior to the bacterin [45].

In order to identify new putative candidate proteins for fish immunization against *A. salmonicida* we checked among the most abundant *A. salmonicida* proteins in SNs and OM proteins associated to pellets to learn whether homologous proteins in pathogenic bacteria were described in the literature to protect the host when inoculated as a subunit vaccine (Additional file 11).

For vaccination, subunits of the T3SS needle have currently been shown to be partial protective immunogens (AopB, AopD, AcrV and AscF homologues). The vaccination of host with T3SS translocon proteins was partially protective in other diseases and might be interesting against *A. salmonicida*. The immunization of a host with recombinant T3SS effector proteins shows variable results and is well-documented in *Yersinia* (Additional file 11). For example, while YopE (homologous to AexT) was not protective in experiments inoculating the whole protein, the N-terminal domain (YopE69-77) was a major protective antigen eliciting CD8 T-cell immunity [46]. This result shows that T3SS effectors can contain protective epitopes that might be promising candidates for vaccination. It could make no sense to vaccinate against proteins that in vivo are directly injected from the bacterial cytoplasm into the host cell and, theoretically, out of reach of the immune system (the single-step conduit model). However, in their “two-step model” for *Yersinia* effector translocation, Akopyan and collaborators [47] have shown that at least YopH (homologous to AopH), YopE (AexT) and YopB/YopD translocators were excreted homogeneously at the bacterial surface (accessible to proteinase K digestion). Such a mechanism of translocation might also occur in *A. salmonicida*, and this might be exploited to vaccinate fish. Moreover, our results clearly show that AopH/Ati2/AexT effectors and AopB/AopD translocators were in the top seven most abundant excreted proteins by wt *A. salmonicida* (Additional file 7). These T3SS components might therefore constitute promising candidates for fish vaccination against furunculosis.

**Figure 7:**
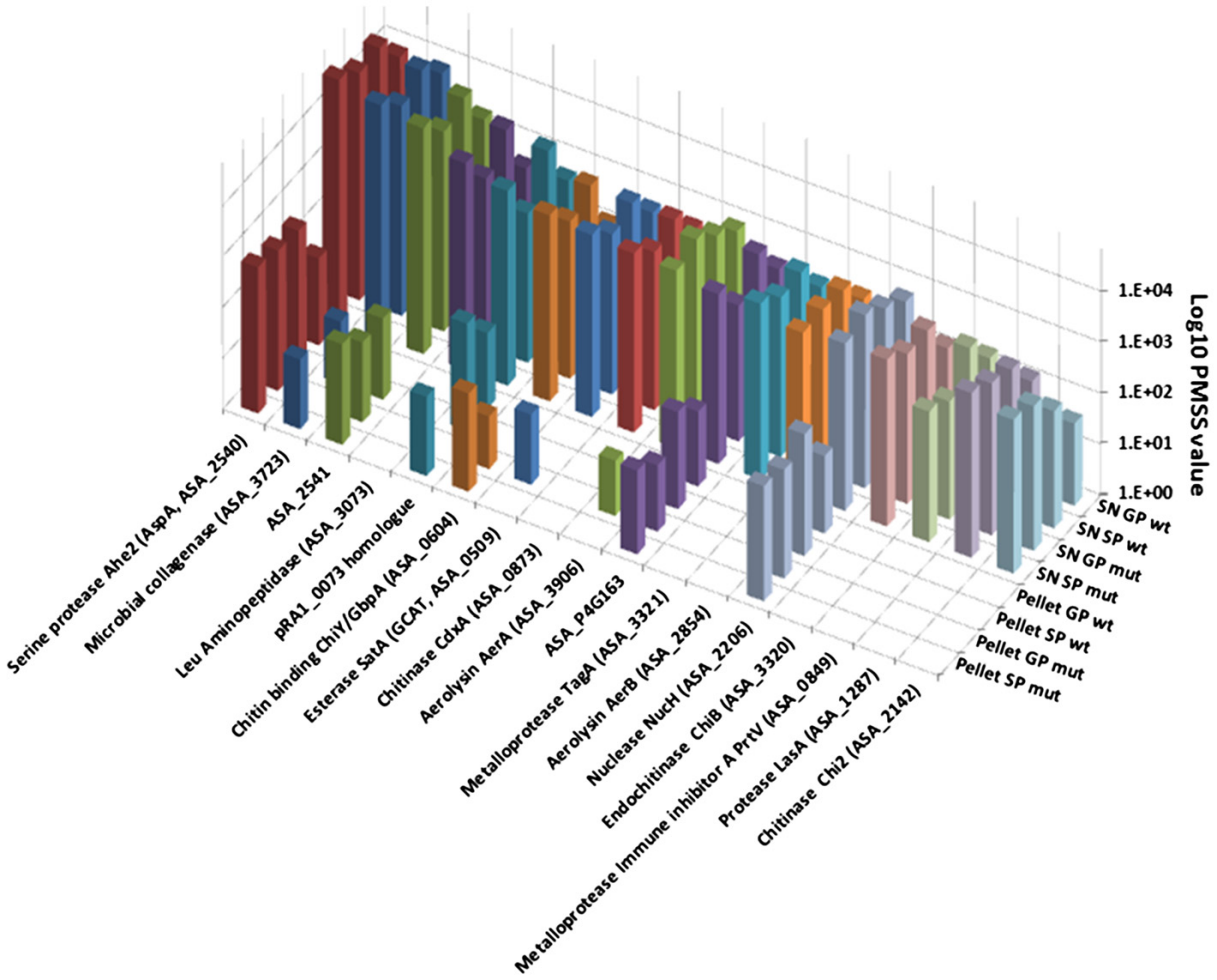
**Quantity values of some proteins abundant in SNs but weak in pellets.** Some of the most secreted proteins that were either not detected or were in a very low amount in bacterial pellets suggest that *A. salmonicida* actively secreted them once they are produced in bacteria. As a result, these proteins are poorly included in pelleted-bacteria killed by formalin solution that are used for vaccination.

Besides the elements of the T3SS, many cytoplasmic proteins that we unexpectedly found in our wt SNs were demonstrated to be immunogenic and recognized by sera from diseased hosts, confirming that they should be extracellularly presented to the immune system by bacteria during the pathogenesis (Additional file 11). Among these putative antigens some show major or partial protective immunogenicity in other pathogenic bacteria and constitute interesting priority candidates for fish vaccine against furunculosis. In decreasing order of quantity in wt SNs we found: EF-Tu, DnaK, CysK, GAPDH (ASA_0759), AhpC, FbaA, HtpG, Pgk, FKBP-type peptidyl-prolyl cis-trans isomerases, OmpAI, 30S ribosomal protein S1, Mdh, ClpP and OmpK40. Another interesting point is that most vaccine assays against furunculosis use bacterial pellets inactivated with formalin, thereby avoiding the extracellular protein (ECP) fraction. However, our results clearly show that some of the most secreted proteins were not detected or were in a very low amount in bacterial pellets, thus suggesting that *A. salmonicida* did not keep these proteins in its cytoplasm but instead actively secreted them. As a result, they are poorly included in bacterins used for vaccination which are pelleted-bacteria killed by formalin solution. In decreasing order of quantity in wt SNs, this was the case for serine protease Ahe2, microbial collagenase ASA_3723, ASA_2541, leucine aminopeptidase ASA_3073, bacterial group Ig-like protein (homologous to pRA1_0073), chitin binding ChiY/GbpA (ASA_0604), GCAT (SatA), chitinase CdxA, Aerolysin A, ASA_P4G163, TagA, AerB, extracellular nuclease NucH, endochitinase ChiB, immune inhibitor A (PrtV, ASA_0849), protease LasA and chitinase Chi2 (Figure 7). While total *A. salmonicida* ECP fraction was demonstrated to be slighty less protective than bacterins in vaccination trials, fish protection against *A. salmonicida* increased with the inoculation of concentrated ECPs [48,49]. The protective antigenic potential of these excreted proteins should therefore not be neglected. Moreover, they were significatively increased in SP SNs, suggesting that they extracellularly accumulated upon secretion. Among OM proteins associated to pellets, GroEL, LamBs, TolC, FadL and BtuB are putative candidates for protective immunization (Additional file 11).

## Conclusions

In this part of our work the analysis by high-throughput proteomics of *A. salmonicida* secretomes gave us the possibility to identify, besides the canonical virulence factors, numerous highly conserved cytoplasmic proteins with putative moonlighting activities whose presence in supernatants was unlikely to be associated to cell lysis. Further investigations will be necessary in order to understand the role of these unexpected extracellular proteins. Major secreted proteins and OMPs that have been successfully used as protective antigens in other diseases were also identified and are promising subunit vaccine candidates to protect fish against furunculosis.

## Methods

### Cell culture and preparation of bacterial supernatants and pellets for LC-MS/MS

*Aeromonas salmonicida* wt and *ΔascV* mutant strains used in this study were characterized in a previous work [7]. To get *A. salmonicida* wt cultures into a maximum T3SS activation state we used JF2267 strain which was freshly reisolated from an experimentally infected dead fish (JF5054). This re-isolated strain was highly virulent, since intraperitoneal inoculation of only 500 cfu per fish was sufficient to induce 70 to 80% of mortality in challenge assays [50]. The *ΔascV* mutant strain JF2747 is considered to have extremely low-virulence because 10^5^ cfu/fish induced no mortality [7], and 10^8^ cfu/fish induced a weak mortality of only 20%.

To precipitate and concentrate proteins from the supernatant of wt and *ΔascV A. salmonicida*, 50 ml of TSB medium were inoculated with 10^9^ bacteria and cultivated at 18°C under shaking (160 rpm) in the presence of protease inhibitors (cOmplete®, Roche Diagnostics). The bacterial growth was stopped during the exponential phase of growth (DO_600_ = ~1.5) and the stationary phase (DO_600_ >2.0). Supernatants were separated from bacterial pellets by centrifugation (6.000 x g, 10 min, 4°C) and filtration through a 0.22 μM Acrodisc filter (low protein binding, PALL Life Sciences). The bacterial pellets were resuspended in 10 ml of PBS, and 250 μL of these solutions were mixed with 250 μL of SDS loading buffer and heated at 100°C for 5 min. To precipitate proteins from supernatants, 12.5 ml of 100% ice-cold trichloroacetic acid were added to the solutions (20% final concentration), then immediately vortexed and incubated overnight on ice. Supernatants were removed and brown protein pellets were suspended and washed several times by centrifugation in ice-cold 100% acetone in 2 ml Eppendorf tubes (low binding protein). Finally, the pellets were dried, diluted in 250 μL of SDS loading buffer (~200 times concentration) and heated at 100°C for 5 min. Proteins were separated in non-adjacent wells (to avoid well to well contamination) on 15% acrylamide SDS-PAGE gels and stained with Coomassie. One run for each of the eight biological conditions (SN and pellet, GP and SP, wt and mutant) was completely sliced from the stacking gel to the buffer front in 20 to 25 bands, and each band was cut into small (~1 mm^3^) cubes for protein in-gel digestion as described elsewhere [51,52]. Peptide sequencing was made on a LTQ Orbitrap XL mass spectrometer (ThermoFisher Scientific, Bremen; Germany) equipped with a Rheos Allegro nano flow system with AFM flow splitting (Flux Instruments, Reinach; Switzerland) and a nano electrospray ion source operated at a voltage of 1.7 kV. Peptide separation was performed on a Magic C18 column (5 μm, 100 Å, 0.075 × 70 mm) using a flow rate of ~400 nL/min and a linear gradient of 5 to 40% acetonitrile in water/0.1% (v/v) formic acid during 60 min.

The mass spectrometry proteomics data were deposited to the ProteomeXchange Consortium (http://proteomecentral.proteomexchange.org) via the PRIDE partner repository [53] with the dataset identifier PXD000429 and DOI 10.6019/PXD000429.

### LC-MS/MS data interpretation

LC-MS/MS data interpretation was made against the current UniProtKB database release (2012_06) of all known *A. salmonicida* protein sequences. Two methods of relative protein quantification were used. The peptide-matching score summation (PMSS) is a label-free technique that assumes ideal scoring for proteins as the summative of the identification scores of their constituent peptides freed upon digestion. A higher score represents a more abundant protein [54]. The EasyProt search algorithm [55] was used for this, as described in [52]. The obtained mass spectrometric raw data were also analyzed with MaxQuant, version 1.2.2.5 [56], and its label-free quantitation (LFQ) algorithms [57] allowed quantitative comparisons. MaxQuant settings were as follows: accepted false discovery rates at peptide, modified peptide and protein level were set at 1% using the reversed sequence database. Carbamidomethylation on Cys was set as a fixed modification. Oxidation of Met, acetylation on protein N-terminus, and phosphorylation on Set/Thr/Tyr were set as variable modifications with a precursor mass tolerance of 6 ppm in the main search, while only oxidation and acetylation with a mass accuracy of 20 ppm was used in the first search. Trypsin cleavage specificity was set at full with a maximum 2 missed cleavages and the allowance of up to three modifications per peptide of length between 6–25 amino acids. Fragment spectra were filtered to the 6 most intense peaks per 100 Da mass windows and searched with a mass tolerance of 0.5 Da. Protein identifications were accepted with at least 2 razor and unique peptide identifications. For label free quantification (LFQ), at least 2 unmodified or acetylated protein N-terminal peptides were required, and matching within a 2 minute time frame between samples was allowed. Only cytoplasmic proteins with significant PMSS and LFQ values in SNs of wt and mutant strains in GP and SP were developed in the text.

### Bioinformatics analysis

Prediction of subcellular localizations was made with Psortb v3.0.2 (http://www.psort.org/psortb/) [13]. Repartition of detected proteins according to Cluster of Orthologous Group was performed with COGnitor (http://www.ncbi.nlm.nih.gov/COG/).

## Abbreviations

GP: Exponential phase of growth; LFQ: Label-free quantitation; PMSS: Peptide-matching score summation; SN: Supernatant; SP: Stationary phase of growth; T3SS: Type-three secretion system; wt: Wild-type.

## Competing interests

The authors declared that they have no competing interests.

## Authors’ contributions

PVB conceived of the study, carried out the experiments, analyzed data from MS, performed bioinformatic analyses and drafted the manuscript. SB-L and MH performed MS experiments and interpretation of MS data. JF helped to draft the manuscript. Authors read and approved the final manuscript.

## Supplementary Material

Additional file 1 **Table.** Raw data of PMSS values from concentrated SNs of wt (A1) and ΔascV mutant (B1) strains in exponential phase of growth. Click here for file

Additional file 2 **Table.** Raw data of PMSS values from pellets of wt (C1) and Δ*ascV* mutant (D1) strains in exponential phase of growth. Click here for file

Additional file 3 **Table.** Raw data of PMSS values from concentrated SNs of wt (A2) and Δ*ascV* mutant (B2) strains in stationary phase of growth. Click here for file

Additional file 4 **Table.** Raw data of PMSS values from pellets of wt (C2) and Δ*ascV* mutant (D2) strains in stationary phase of growth. Click here for file

Additional file 5 **Table.** Raw data of LFQ values (MaxQuant) from SNs. A1 and A2 = wt SNs in GP and SP; B1 and B2 = mutant SNs in GP and SP. Click here for file

Additional file 6 **Table.** Raw data of LFQ values (MaxQuant) from pellets. C1 and C2 = wt pellets in GP and SP; D1 and D2 = mutant pellets in GP and SP. Click here for file

Additional file 7 **Table.** PMSS, LFQ values, ratios, PEP values, subcellular localization, secretion system signals for each protein identified in SNs and pellets of wt and mutant strains in GP and SP. Column B: Proteins names. Red = T3SS components; dark red = secondary virulence factors; light red = putative secondary virulence factors; yellow = proteins specific of JF2267 or B526; mauve = multidrug resistance-associated proteins; orange = ABC transporters; light green = proteins associated to flagella, pili, T4SS; dark blue = phage proteins; light blue = cytoplasmic moonlighting proteins present in SNs; grey = T5SS; light pink T6SS, pink: transposases. Column E: A449 Loci. Grey = genes conserved among *Aeromonas* sp.; white = genes shared with at least one other *Aeromonas* species; green = genes specific of *A. salmonicida*; yellow = genes specific of *A. salmonicida* JF2267 and B526; pink = transposases. Click here for file

Additional file 8 **Table.** cytoplasmic proteins abundantly detected in *A. salmonicida* SNs and identified in the secretome of other bacteria. The moonlighting activity is mentioned (when it is known). Click here for file

Additional file 9 **Table.***A. salmonicida* proteins associated to the outer membrane identified by Ebanks and collaborators [19] and detected in our SNs. The Table shows proteins associated to the *A. salmonicida* OM identified by Ebanks and collaborators and detected in our SNs and pellets. In grey are indicated proteins with moonlighting activity. Click here for file

Additional file 10 **Table.***Pseudomonas aeruginosa* proteins detected by Choi and collaborators in outer membrane vesicles [43] and their homologues in *A. salmonicida* subsp*. salmonicida.* In green and gray, proteins respectively detected in *A. salmonicida* SNs and pellets by our MS analysis and having homologues in *P. aeruginosa* OMVs. In white, OMV proteins specific to *P. aeruginosa*. Click here for file

Additional file 11 **Table.***A. salmonicida* proteins identified in SNs or associated to the OM that have antigenic homologues in other bacteria and constitute candidates for subunit vaccine. Click here for file
