# Supplementary material for: The Aeromonas salmonicida subsp. salmonicida exoproteome: global analysis, moonlighting proteins and putative antigens for vaccination against furunculosis
Source: Proteome Sci. 2013 Oct 15;11:44. doi: 10.1186/1477-5956-11-44 (PMC3826670; doi:10.1186/1477-5956-11-44)
Supplement: Additional file 8 — Table. cytoplasmic proteins abundantly detected in A. salmonicida SNs and identified in the secretome of other bacteria. The moonlighting activity is mentioned (when it is known). [file 1477-5956-11-44-S8.doc]

| Protein | A449 locus | Name | Identification of homologous protein in the secretome of other bacteria and moonlighting activity (if known) | | Ref. |
| --- | --- | --- | --- | --- | --- |
|  |  |  | Organism | Moonlighting activity |  |
| EF-Tu | ASA_0275  ASA_0293 | Elongation factor Tu | *Pseudomonas aeruginosa* | Binding to factor H and plasminogen  Immune evasion | [1] |
|  |  |  | *Rhizobium etli* |  | [2] |
|  |  |  | *Erwinia chrysanthemi* |  | [3] |
|  |  |  | *Salmonella typhimurium* |  | [4,5] |
|  |  |  | *Erwinia carotovora* |  | [6] |
|  |  |  | *Bacillus anthracis* |  | [7] |
|  |  |  | *Enterococcus faecalis* |  | [8] |
|  |  |  | *Streptococcus suis* |  | [9] |
| EF-G | ASA_0292 | Elongation factor G | *Rhizobium etli* |  | [2] |
|  |  |  | *Vibrio cholerae* |  | [10] |
|  |  |  | *Salmonella typhimurium* |  | [4,5] |
|  |  |  | *Bacillus anthracis* |  | [7] |
|  |  |  | *Burkholderia cepacia* |  | [11] |
| EF-Ts | ASA_3159 | Elongation factor Ts | *Rhizobium etli* |  | [2] |
|  |  |  | *Salmonella typhimurium* |  | [4] |
|  |  |  | *Erwinia carotovora* |  | [6] |
|  |  |  | *Bacillus anthracis* |  | [7] |
|  |  |  | *Burkholderia cepacia* |  | [11] |
| TypA | ASA_4119 | GTP-binding elongation factor protein | *Rhizobium etli* |  | [2] |
|  |  |  | *Erwinia carotovora* |  | [6] |
| RpsA | ASA_1768 | 30S ribosomal protein S1 | *Rhizobium etli* |  | [2] |
|  |  |  | *Salmonella typhimurium* |  | [4] |
|  |  |  | *Erwinia carotovora* |  | [6] |
|  |  |  | *Streptococcus suis* |  | [9] |
| RpsE | ASA_4070 | 30S ribosomal protein S5 | *Rhizobium etli* |  | [2] |
|  |  |  | *Salmonella typhimurium* |  | [5] |
|  |  |  | *Erwinia carotovora* |  | [6] |
|  |  |  | *Bacillus anthracis* |  | [7] |
| RpsH | ASA_4073 | 30S ribosomal protein S8 | *Rhizobium etli* |  | [2] |
|  |  |  | *Salmonella typhimurium* |  | [5] |
|  |  |  | *Streptococcus suis* |  | [9] |
| RpsP | ASA_0666 | 30S ribosomal protein S16 | *Salmonella typhimurium* |  | [5] |
| RplA | ASA_0280 | 50S ribosomal protein L1 | *Rhizobium etli* |  | [2] |
|  |  |  | *Salmonella typhimurium* |  | [4,5] |
|  |  |  | *Erwinia carotovora* |  | [6] |
|  |  |  | *Bacillus anthracis* |  | [7] |
|  |  |  | *Streptococcus suis* |  | [9] |
| RplC | ASA_4087 | 50S ribosomal protein L3 | *Rhizobium etli* |  | [2] |
|  |  |  | *Salmonella typhimurium* |  | [4] |
|  |  |  | *Erwinia carotovora* |  | [6] |
|  |  |  | *Streptococcus suis* |  | [9] |
| RplF | ASA_4072 | 50S ribosomal protein L6 | *Rhizobium etli* |  | [2] |
|  |  |  | *Salmonella typhimurium* |  | [4,5] |
|  |  |  | *Erwinia carotovora* |  | [6] |
|  |  |  | *Bacillus anthracis* |  | [7] |
|  |  |  | *Enterococcus faecalis* |  | [8] |
|  |  |  | *Streptococcus suis* |  | [9] |
| RplK | ASA_0279 | 50S ribosomal protein L11 | *Rhizobium etli* |  | [2] |
|  |  |  | *Salmonella typhimurium* |  | [5] |
|  |  |  | *Bacillus anthracis* |  | [7] |
|  |  |  | *Francisella tularensis* |  | [12] |
| AlaS | ASA_3811 | Alanyl tRNA synthetase | *Rhizobium etli* |  | [2] |
|  |  |  | *Salmonella typhimurium* |  | [4] |
|  |  |  | *Erwinia carotovora* |  | [6] |
|  |  |  | *Bacillus anthracis* |  | [7] |
| HtpG  (HSP90) | ASA_1826 | Heat shock protein G | *Streptococcus suis* |  | [13] |
|  |  |  | *Rhizobium etli* |  | [2] |
|  |  |  | *Porphyromonas gingivalis* | Chemokine CXCL8 induction in human monocytic and microvascular vein endothelial cells | [14] |
|  |  |  | *Erwinia carotovora* |  | [6] |
| DnaK  (HSP70) | ASA_2996 | Heat shock protein 70 | *Bifidobacterium animalis subsp. lactis* | Binding to plasminogen | [15] |
|  |  |  | *Mycobacterium tuberculosis* | Binding to plasminogen  Stimulates CD8 lymphocyte chemokine production  Stimulates monocyte chemokine synthesis and dendritic cell maturation by binding CD40 and CCR5  Increase cytokine production in macrophages to modulate immunity | [16-20] |
|  |  |  | *Coxiella burnetii* |  | [21] |
|  |  |  | *Francisella tularensis* | Dendritic cell stimulation through TLR4 | [22] |
|  |  |  | *E. coli EPEC* | Binding to host sulphogalactosylceramide | [23] |
|  |  |  | *Legionella pneumophila* |  | [24] |
|  |  |  | *Haemophilus influenzae* | Binding to sulphated galactolipids | [25] |
|  |  |  | *Lactobacillus plantarum* |  | [26] |
|  |  |  | *Lactobacillus lactis* | Binding to yeast mannan | [27] |
|  |  |  | *Neisseria meningitidis* | Binding to plasminogen | [28] |
|  |  |  | *Rhizobium etli* |  | [2] |
|  |  |  | *Salmonella typhimurium* |  | [4] |
|  |  |  | *Erwinia carotovora* |  | [6] |
|  |  |  | *Bacillus anthracis* |  | [7] |
|  |  |  | *Burkholderia cepacia* |  | [11] |
|  |  |  | *Enterococcus faecalis* |  | [8] |
|  |  |  | *Francisella tularensis* |  | [12] |
|  |  |  | *Streptococcus suis* | Adhesion to host cells | [9] |
| Tig | ASA_1888 |  | *Rhizobium etli* |  | [2] |
|  |  |  | *Salmonella typhimurium* |  | [4] |
|  |  |  | *Erwinia carotovora* |  | [6] |
|  |  |  | *Bacillus anthracis* |  | [7] |
|  |  |  | *Streptococcus suis* |  | [9] |
| PpiC | ASA_1247 | FKBP-type peptidyl-prolyl cis-trans isomerase | *Helicobacter pylori* | Induction of gastric epithelial cells apoptosis in a TLR4- and apoptosis signal-regulating kinase 1-dependent manner  Activation of monocyte IL-6 synthesis | [29,30] |
| PpiB | ASA_1614 |  |  |  |  |
| PpiA | ASA_3203 |  | *Histoplasma capsulatum* | Binding to dendritic cell VLA-5 | [31] |
|  |  |  | *Bacillus anthracis* |  | [7] |
|  |  |  | *Legionella pneumophila* | Binding to various collagens  MIP: Macrophage infectivity potentiator | [32-35] |
|  |  |  | *Neisseria gonorrhoeae* | Persistence in macrophages | [36] |
|  |  |  | *Rickettsia prowazekii* | ? | [37] |
|  |  |  | *Streptococcus pneumoniae* | ? | [38] |
|  |  |  | *Vibrio cholerae* |  | [10] |
|  |  |  | *Erwinia carotovora* |  | [6] |
|  |  |  | *Rhizobium etli* |  | [2] |
| GAPDH | ASA_0759 | Glyceraldehyde-3-phosphate dehydrogenase  Homologous to epd (ASA_3506) GapA (ASA_0946) | *Streptococcus pyogenes S. pneumonia*  *S. agalactiae*  *S. suis* | Binding to lysozyme, cytoskeletal proteins, fibronectin, plasminogen, albumin  Adherence to host cells (uPAR/CD87) and antiphagocytic activity  Inhibition of complement (C5a), blocking neutrophil chemotaxis and hydrogen peroxide production  B lymphocyte-modulatory activity | [9,39-45] |
|  |  |  | *Escherichia coli EHEC and EPEC* | Binding to plasminogen and fibrinogen  NAD-ribosylating activity | [46] |
|  |  |  | *Mycoplasma genitalium* | Binding to mucin | [47] |
|  |  |  | *Lactobacillus plantarum* | Binding to gastric mucin and intestinal epithelial cell line  Binding to human ABO blood group antigens | [48]  [49] |
|  |  |  | *Staphylococcus aureus* | Receptor for transferrin | [50] |
|  |  |  | *Rhizobium etli* |  | [2] |
|  |  |  | *Salmonella typhimurium* |  | [4] |
|  |  |  | *Erwinia carotovora* |  | [6] |
|  |  |  | *Bacillus anthracis* | Binding to plasminogen | [7,51] |
|  |  |  | *Enterococcus faecalis* |  | [8] |
|  |  |  | *Francisella tularensis* |  | [12] |
|  |  |  | *Burkholderia cepacia* |  | [11] |
| Eno | ASA_3475 | Enolase | *Aeromonas hydrophila* | Binding to plasminogen | [52] |
|  |  |  | *Lactobacillus plantarum* | Binding to fibronectin | [53] |
|  |  |  | *Mycoplasma fermentans* | Binding to plasminogen | [54] |
|  |  |  | *Neisseria meningitidis* | Binding to plasminogen | [28] |
|  |  |  | *Borrelia burgdorferi* | Binding to plasminogen | [55] |
|  |  |  | *Staphylococcus aureus* | Binding to laminin | [56] |
|  |  |  | *Streptococcus gordonii*  *S. mutans*  *S. pneumonia*  *S. suis*  *S. pyogenes* | Binding to salivary mucin (MUC7, MG2), plasminogen and fibronectin  Adhesion to host cells  Complement evasion via C4BP-binding | [9,57-61] |
|  |  |  | *Rhizobium etli* |  | [2] |
|  |  |  | *Salmonella typhimurium* |  | [4,5] |
|  |  |  | *Bacillus anthracis* |  | [7] |
|  |  |  | *Burkholderia cepacia* |  | [11] |
|  |  |  | *Erwinia carotovora* |  | [6] |
|  |  |  | *Enterococcus faecalis* |  | [8] |
| FbaA | ASA_3504 | Fructose-biphosphate aldolase class II | *S. pneumoniae* | Binding to host cadherin (Flamingo) | [62] |
|  |  |  | *Rhizobium etli* |  | [2] |
|  |  |  | *N. meningitidis* | Adherence to host cells | [63] |
|  |  |  | *Salmonella typhimurium* |  | [4] |
|  |  |  | *Erwinia carotovora* |  | [6] |
|  |  |  | *Bacillus anthracis* |  | [7] |
| TpiA | ASA_2232 | Triose phosphate isomerase | *S. aureus* | Adhesion to mannans of Cryptococcus neoformans for contact-mediated killing | [64-66] |
|  |  |  | *Salmonella typhimurium* |  | [4] |
|  |  |  | *Erwinia carotovora* |  | [6] |
|  |  |  | *Francisella tularensis* |  | [12] |
|  |  |  | *Burkholderia cepacia* |  | [11] |
| Pgk | ASA_3505 | Phosphoglycerate kinase | *Oral streptococci*  *Group B streptococci* | Binding to plasminogen  Binding to cellular actin | [67,68] |
|  |  |  | *Salmonella typhimurium* |  | [4] |
|  |  |  | *Bacillus anthracis* |  | [7] |
|  |  |  | *Burkholderia cepacia* |  | [11] |
|  |  |  | *Erwinia carotovora* |  | [6] |
|  |  |  | *Enterococcus faecalis* |  | [8] |
| TktA | ASA_1202 | Transketolase A | *Rhizobium etli* |  | [2] |
|  |  |  | *Salmonella typhimurium* |  | [4] |
|  |  |  | *Bacillus anthracis* |  | [7] |
|  |  |  | *Burkholderia cepacia* |  | [11] |
| Pta | ASA_3402 | Phosphate acetyltransferase | *Erwinia carotovora* |  | [6] |
| AckA | ASA_3401 | Acetate kinase A | *Erwinia carotovora* |  | [6] |
|  |  |  | *Bacillus anthracis* |  | [7] |
| AcnB | ASA_0427 | Aconitate hydratase B | *Mycobacterium tuberculosis* | Iron-dependent RNA-binding activity | [69] |
|  |  |  | *Erwinia carotovora* |  | [6] |
|  |  |  | *Bacillus anthracis* |  | [7] |
| Mdh | ASA_0659 | Malate dehydrogenase | *Rhizobium etli* |  | [2] |
|  |  |  | *Vibrio cholerae* |  | [10] |
|  |  |  | *Erwinia carotovora* |  | [6] |
|  |  |  | *Bacillus anthracis* |  | [7] |
| AhpC | ASA_2627 | Alkyl hydroperoxide reductase C (Peroxiredoxin) (homologue ASA_2851) | *Vibrio cholerae* |  | [10] |
|  |  |  | *Salmonella typhimurium* |  | [4] |
|  |  |  | *Erwinia carotovora* |  | [6] |
|  |  |  | *Bacillus anthracis* |  | [7] |
|  |  |  | *Francisella tularensis* |  | [12] |
|  |  |  | *Burkholderia cepacia* |  | [11] |
|  |  |  | *Enterococcus faecalis* | Resistance to oxidative stress | [70] |
| Tpx | ASA_0630 | Thiol peroxidase (Peroxiredoxin) | *Neisseria meningitidis* | Binding to plasminogen | [28] |
|  |  |  | *Salmonella typhimurium* |  | [4] |
|  |  |  | *Erwinia carotovora* |  | [6] |
|  |  |  | *Bacillus anthracis* |  | [7] |
|  |  |  | *Mycobacterium tuberculosis* | Resistance to oxidative and nitrosative stress | [71] |
|  |  |  | *Enterococcus faecalis* | Resistance to oxidative stress | [70] |
| SodB | ASA_1375 | Superoxide dismutase | *Rhizobium etli* |  | [2] |
|  |  |  | *Salmonella typhimurium* |  | [4] |
|  |  |  | *Francisella tularensis* |  | [12] |
| Pnp | ASA_1014 | Polyribonucleotide nucleotidyltransferase | *Rhizobium etli* |  | [2] |
|  |  |  | *Vibrio cholerae* |  | [10] |
|  |  |  | *Erwinia carotovora* |  | [6] |
|  |  |  | *Bacillus anthracis* |  | [7] |
|  |  |  | *Burkholderia cepacia* |  | [11] |
|  |  |  | *Streptococcus suis* |  | [9] |
| Adk | ASA_1827 | Adenylate kinase | *Salmonella typhimurium* |  | [5] |
| Upp | ASA_1511 | Uracil phosphoribosyltransferase | *Bacillus anthracis* |  | [7] |

**References:**

1. Kunert A, Losse J, Gruszin C, Huhn M, Kaendler K, Mikkat S, Volke D, Hoffmann R, Jokiranta TS, Seeberger H et al.: **Immune evasion of the human pathogen *Pseudomonas aeruginosa*: elongation factor Tuf is a factor H and plasminogen binding protein.** *J Immunol* 2007, **179:**2979-2988.

2. Meneses N, Mendoza-Hernandez G, Encarnacion S: **The extracellular proteome of *Rhizobium etli* CE3 in exponential and stationary growth phase.** *Proteome Sci* 2010, **8**.

3. Kazemi-Pour N, Condemine G, Hugouvieux-Cotte-Pattat N: **The secretome of the plant pathogenic bacterium *Erwinia chrysanthemi*.** *Proteomics* 2004, **4:**3177-3186.

4. Sherry AE, Inglis NF, Stevenson A, Fraser-Pitt D, Everest P, Smith DGE, Roberts M: **Characterisation of proteins extracted from the surface of *Salmonella Typhimurium* grown under SPI-2-inducing conditions by LC-ESI/MS/MS sequencing.** *Proteomics* 2011, **11:**361-370.

5. Niemann GS, Brown RN, Gustin JK, Stufkens A, Shaikh-Kidwai AS, Li J, McDermott JE, Brewer HM, Schepmoes A, Smith RD et al.: **Discovery of novel secreted virulence factors from *Salmonella enterica* serovar *typhimurium* by proteomic analysis of culture supernatants.** *Infect Immun* 2011, **79:**33-43.

6. Coulthurst SJ, Lilley KS, Hedley PE, Liu H, Toth IK, Salmond GPC: **DsbA plays a critical and multifaceted role in the production of secreted virulence factors by the phytopathogen *Erwinia carotovora* subsp *atroseptica*.** *J Biol Chem* 2008, **283:**23739-23753.

7. Walz A, Mujer CV, Connolly JP, Alefantis T, Chafin R, Dake C, Whittington J, Kumar SP, Khan AS, DelVecchio VG: ***Bacillus anthracis* secretome time course under host-simulated conditions and identification of immunogenic proteins.** *Proteome Sci* 2007, **5:**11.

8. Pessione A, Lamberti C, Cocolin L, Campolongo S, Grunau A, Giubergia S, Eberl L, Riedel K, Pessione E: **Different protein expression profiles in cheese and clinical isolates of *Enterococcus faecalis* revealed by proteomic analysis.** *Proteomics* 2012, **12:**431-447.

9. Chen B, Zhang AD, Xu ZM, Li R, Chen HC, Jin ML: **Large-scale identification of bacteria-host crosstalk by affinity chromatography: capturing the interactions of *Streptococcus suis* proteins with host cells.** *J Proteome Res* 2011, **10:**5163-5174.

10. Sikora AE, Zielke RA, Lawrence DA, Andrews PC, Sandkvist M: **Proteomic analysis of the *Vibrio cholerae* type II secretome reveals new proteins, including three related serine proteases.** *J Biol Chem* 2011, **286:**16555-16566.

11. Mariappan V, Vellasamy KM, Hashim OH, Vadivelu J: **Profiling of *Burkholderia cepacia* secretome at mid-logarithmic and early-stationary phases of growth.** *Plos One* 2011, **6**.

12. Konecna K, Hernychova L, Reichelova M, Lenco J, Klimentova J, Stulik J, Macela A, Alefantis T, DelVecchio VG: **Comparative proteomic profiling of culture filtrate proteins of less and highly virulent *Francisella tularensis* strains.** *Proteomics* 2010, **10:**4501-4511.

13. Wu ZF, Zhang W, Lu CP: **Comparative proteome analysis of secreted proteins of *Streptococcus suis* serotype 9 isolates from diseased and healthy pigs.** *Microb Pathog* 2008, **45:**159-166.

14. Shelburne CE, Coopamah MD, Sweier DG, An FY, Lopatin DE: **HtpG, the *Porphyromonas gingivalis* HSP-90 homologue, induces the chemokine CXCL8 in human monocytic and microvascular vein endothelial cells.** *Cell Microbiol* 2007, **9:**1611-1619.

15. Candela M, Centanni M, Fiori J, Biagi E, Turroni S, Orrico C, Bergmann S, Hammerschmidt S, Brigidi P: **DnaK from *Bifidobacterium animalis* subsp *lactis* is a surface-exposed human plasminogen receptor upregulated in response to bile salts.** *Microbiology - SGM* 2010, **156:**1609-1618.

16. Lehner T, Bergmeier LA, Wang YF, Tao L, Sing M, Spallek R, van der Zee R: **Heat shock proteins generate beta-chemokines which function as innate adjuvants enhancing adaptive immunity.** *Eur J Immunol* 2000, **30:**594-603.

17. Wang YF, Kelly CG, Karttunen JT, Whittall T, Lehner PJ, Duncan L, MacAry P, Younson JS, Singh M, Oehlmann W et al.: **CD40 is a cellular receptor mediating mycobacterial heat shock protein 70 stimulation of CC-chemokines.** *Immunity* 2001, **15:**971-983.

18. Xolalpa W, Vallecillo AJ, Lara M, Mendoza-Hernandez G, Comini M, Spalle R, Singh M, Espitia C: **Identification of novel bacterial plasminogen-binding proteins in the human pathogen *Mycobacterium tuberculosis*.** *Proteomics* 2007, **7:**3332-3341.

19. Hickey TBM, Thorson LM, Speert DP, Daffe M, Stokes RW: ***Mycobacterium tuberculosis* Cpn60.2 and DnaK are located on the bacterial surface, where Cpn60.2 facilitates efficient bacterial association with macrophages.** *Infect Immun* 2009, **77:**3389-3401.

20. Retzlaff C, Yamamoto Y, Hoffman PS, Friedman H, Klein TW: **Bacterial heat shock proteins directly induce cytokine mRNA and interleukin-1 secretion in macrophage cultures.** *Infect Immun* 1994, **62:**5689-5693.

21. Macellaro A, Tujulin E, Hjalmarsson K, Norlander L: **Identification of a 71-kilodalton surface-associated Hsp70 homologue in *Coxiella burnetii*.** *Infect Immun* 1998, **66:**5882-5888.

22. Ashtekar AR, Zhang P, Katz J, Deivanayagam CC, Rallabhandi P, Vogel SN, Michalek SM: **TLR4-mediated activation of dendritic cells by the heat shock protein DnaK from *Francisella tularensis*.** *J Leukoc Biol* 2008, **84:**1434-1446.

23. de Jesus MC, Urban AA, Marasigan ME, Foster DEB: **Acid and bile-salt stress of enteropathogenic *Escherichia coli* enhances adhesion to epithelial cells and alters glycolipid receptor binding specificity.** *J Infect Dis* 2005, **192:**1430-1440.

24. Hoffman PS, Garduno RA: **Surface-associated heat shock proteins of *Legionella pneumophila* and *Helicobacter pylori*: roles in pathogenesis and immunity.** *Infect Dis Obstet Gynecol* 1999, **7:**58-63.

25. Hartmann E, Lingwood CA, Reidl J: **Heat-inducible surface stress protein (Hsp70) mediates sulfatide recognition of the respiratory pathogen *Haemophilus influenzae*.** *Infect Immun* 2001, **69:**3438-3441.

26. Saad N, Urdaci M, Vignoles C, Chaignepain S, Tallon R, Schmitter JM, Bressollier P: ***Lactobacillus plantarum* 299v surface-bound GAPDH: a new insight into enzyme cell walls location.** *J Microbiol Biotechnol* 2009, **19:**1635-1643.

27. Katakura Y, Sano R, Hashimoto T, Ninomiya K, Shioya S: **Lactic acid bacteria display on the cell surface cytosolic proteins that recognize yeast mannan.** *Appl Microbiol Biotechnol* 2010, **86:**319-326.

28. Knaust A, Weber MVR, Hammerschmidt S, Bergmann S, Frosch M, Kurzai O: **Cytosolic proteins contribute to surface plasminogen recruitment of *Neisseria meningitidis*.** *J Bact* 2007, **189:**3246-3255.

29. Basak C, Pathak SK, Bhattacharyya A, Pathak S, Basu J, Kundu M: **The secreted peptidyl prolyl cis,trans-isomerase HP0175 of *Helicobacter pylori* induces apoptosis of gastric epithelial cells in a TLR4- and apoptosis signal-regulating kinase 1-dependent manner.** *J Immunol* 2005, **174:**5672-5680.

30. Pathak SK, Basu S, Bhattacharyya A, Pathak S, Banerjee A, Basu J, Kundu M: **TLR4-dependent NF-*kappa* B activation and mitogen- and stress-activated protein kinase 1-triggered phosphorylation events are central to *Helicobacter pylori* peptidyl prolyl cis-, trans-isomerase (HP0175)-mediated induction of IL-6 release from macrophages.** *J Immunol* 2006, **177:**7950-7958.

31. Gomez FJ, Pilcher-Roberts R, Alborzi A, Newman SL: ***Histoplasma capsulatum* cyclophilin A mediates attachment to dendritic cell VLA-5.** *J Immunol* 2008, **181:**7106-7114.

32. Helbig JH, Luck PC, Steinert M, Jacobs E, Witt M: **Immunolocalization of the Mip protein of intracellularly and extracellularly grown *Legionella pneumophila*.** *Let Appl Microbiol* 2001, **32:**83-88.

33. Helbig JH, Konig B, Knospe H, Bubert B, Yu C, Luck CP, Riboldi-Tunnicliffe A, Hilgenfeld R, Jacobs E, Hacker J et al.: **The PPIase active site of *Legionella pneumophila* Mip protein is involved in the infection of eukaryotic host cells.** *Biol Chem* 2003, **384:**125-137.

34. Wagner C, Khan AS, Kamphausen T, Schmausser B, Unal C, Lorenz U, Fischer G, Hacker J, Steinert M: **Collagen binding protein Mip enables *Legionella pneumophila* to transmigrate through a barrier of NCI-H292 lung epithelial cells and extracellular matrix.** *Cell Microbiol* 2007, **9:**450-462.

35. Kohler R, Fanghanel J, Konig B, Luneberg E, Frosch M, Rahfeld JU, Hilgenfeld R, Fischer G, Hacker J, Steinert M: **Biochemical and functional analyses of the Mip protein: Influence of the N-terminal half and of peptidylprolyl isomerase activity on the virulence of *Legionella pneumophila*.** *Infect Immun* 2003, **71:**4389-4397.

36. Leuzzi R, Serino L, Scarselli M, Savino S, Fontana MR, Monaci E, Taddei A, Fischer G, Rappuoli R, Pizza M: **Ng-MIP, a surface-exposed lipoprotein of *Neisseria gonorrhoeae*, has a peptidyl-prolyl cis/trans isomerase (PPIase) activity and is involved in persistence in macrophages.** *Mol Microbiol* 2005, **58:**669-681.

37. Emelyanov VV, Loukianov EV: **A 29.5 KDa heat-modifiable major outer membrane protein of *Rickettsia prowazekii*, putative virulence factor, is a peptidyl-prolyl cis/trans isomerase.** *Iubmb Life* 2004, **56:**215-219.

38. Hermans PWM, Adrian PV, Albert C, Estevao S, Hoogenboezem T, Luijendijk IHT, Kamphausen T, Hammerschmidt S: **The streptococcal lipoprotein rotamase A (SlrA) is a functional peptidyl-prolyl isomerase involved in pneumococcal colonization.** *J Biol Chem* 2006, **281:**968-976.

39. Boel G, Jin H, Pancholi V: **Inhibition of cell surface export of group A streptococcal anchorless surface dehydrogenase affects bacterial adherence and antiphagocytic properties.** *Infect Immun* 2005, **73:**6237-6248.

40. Pancholi V, Fischetti VA: **A major surface protein on group-A *Streptococci* is a glyceraldehyde-3-phosphate-dehydrogenase with multiple binding-activity.** *J Exp Med* 1992, **176:**415-426.

41. Winram SB, Lottenberg R: **The plasmin-binding protein Plr of group A *streptococci* is identified as glyceraldehyde-3-phosphate dehydrogenase.** *Microbiology -UK* 1996, **142:**2311-2320.

42. Jin H, Song YP, Boel G, Kochar J, Pancholi V: **Group A streptococcal surface GAPDH, SDH, recognizes uPAR/CD87 as its receptor on the human pharyngeal cell and mediates bacterial adherence to host cells.** *J Mol Biol* 2005, **350:**27-41.

43. Bergmann S, Rohde M, Hammerschmidt S: **Glyceraldehyde-3-phosphate dehydrogenase of *Streptococcus pneumoniae* is a surface-displayed plasminogen-binding protein.** *Infect Immun* 2004, **72:**2416-2419.

44. Madureira P, Baptista M, Vieira M, Magalhaes V, Camelo A, Oliveira L, Ribeiro A, Tavares D, Trieu-Cuot P, Vilanova M et al.: ***Streptococcus agalactiae* GAPDH is a virulence-associated immunomodulatory protein.** *J Immunol* 2007, **178:**1379-1387.

45. Quessy S, Busque P, Higgins R, Jacques M, Dubreuil JD: **Description of an albumin binding activity for *Streptococcus suis* serotype 2.** *FEMS Microbiol Let* 1997, **147:**245-250.

46. Egea L, Aguilera L, Gimenez R, Sorolla MA, Aguilar J, Badia J, Baldoma L: **Role of secreted glyceraldehyde-3-phosphate dehydrogenase in the infection mechanism of enterohemorrhagic and enteropathogenic *Escherichia coli*: interaction of the extracellular enzyme with human plasminogen and fibrinogen.** *Int J Biochem Cell B* 2007, **39:**1190-1203.

47. Alvarez RA, Blaylock MW, Baseman JB: **Surface localized glyceraldehyde-3-phosphate dehydrogenase of *Mycoplasma genitalium* binds mucin.** *Mol Microbiol* 2003, **48:**1417-1425.

48. Kinoshita H, Uchida H, Kawai Y, Kawasaki T, Wakahara N, Matsuo H, Watanabe M, Kitazawa H, Ohnuma S, Miura K et al.: **Cell surface *Lactobacillus plantarum* LA 318 glyceraldehyde-3-phosphate dehydrogenase (GAPDH) adheres to human colonic mucin.** *J Appl Microbiol* 2008, **104:**1667-1674.

49. Ramiah K, van Reenen CA, Dicks LMT: **Surface-bound proteins of *Lactobacillus plantarum* 423 that contribute to adhesion of Caco-2 cells and their role in competitive exclusion and displacement of *Clostridium sporogenes* and *Enterococcus faecalis*.** *Res Microbiol* 2008, **159:**470-475.

50. Modun B, Morrissey J, Williams P: **The staphylococcal transferrin receptor: a glycolytic enzyme with novel functions.** *Trends Microbiol* 2000, **8:**231-237.

51. Matta SK, Agarwal S, Bhatnagar R: **Surface localized and extracellular glyceraldehyde-3-phosphate dehydrogenase of *Bacillus anthracis* is a plasminogen binding protein.** *Biochim Biophys Acta-Proteins and Proteomics* 2010, **1804:**2111-2120.

52. Sha J, Erova TE, Alyea RA, Wang SF, Olano JP, Pancholi V, Chopra AK: **Surface-expressed enolase contributes to the pathogenesis of clinical isolate SSU of *Aeromonas hydrophila*.** *J Bact* 2009, **191:**3095-3107.

53. Castaldo C, Vastano V, Siciliano RA, Candela M, Vici M, Muscariello L, Marasco R, Sacco M: **Surface displaced *alfa*-enolase of *Lactobacillus plantarum* is a fibronectin binding protein.** *Microb Cell Fact* 2009, **8**.

54. Yavlovich A, Rechnitzer H, Rottem S: ***Alpha*-enolase resides on the cell surface of *Mycoplasma fermentans* and binds plasminogen.** *Infect Immun* 2007, **75:**5716-5719.

55. Floden AM, Watt JA, Brissette CA: ***Borrelia burgdorferi* enolase is a surface-exposed plasminogen binding protein.** *Plos One* 2011, **6**.

56. Carneiro CRW, Postol E, Nomizo R, Reis LFL, Brentani RR: **Identification of enolase as a laminin-binding protein on the surface of *Staphylococcus aureus*.** *Microbes Infect* 2004, **6:**604-608.

57. Kesimer M, Kilic N, Mehrotra R, Thornton DJ, Sheehan JK: **Identification of salivary mucin MUC7 binding proteins from *Streptococcus gordonii*.** *BMC Microbiol* 2009, **9**.

58. Ge JP, Catt DM, Gregory RL: ***Streptococcus mutans* surface *alpha*-enolase binds salivary mucin MG2 and human plasminogen.** *Infect Immun* 2004, **72:**6748-6752.

59. Kolberg J, Aase A, Bergmann S, Herstad TK, Rodal G, Frank R, Rohde M, Hammerschmidt S: ***Streptococcus pneumoniae* enolase is important for plasminogen binding despite low abundance of enolase protein on the bacterial cell surface.** *Microbiology - SGM* 2006, **152:**1307-1317.

60. Pancholi V, Fischetti VA: ***Alpha*-enolase, a novel strong plasmin(ogen) binding protein on the surface of pathogenic *streptococci*.** *J Biol Chem* 1998, **273:**14503-14515.

61. Agarwal V, Hammerschmidt S, Malm S, Bergmann S, Riesbeck K, Blom AM: **Enolase of *Streptococcus pneumoniae* binds human complement inhibitor C4b-binding protein and contributes to complement evasion.** *J Immunol* 2012, **189:**3575-3584.

62. Blau K, Portnoi M, Shagan M, Kaganovich A, Rom S, Kafka D, Caspi VC, Porgador A, Givon-Lavi N, Gershoni JM et al.: **Flamingo cadherin: a putative host receptor for *Streptococcus pneumoniae*.** *J Infect Dis* 2007, **195:**1828-1837.

63. Tunio SA, Oldfield NJ, Berry A, Ala'Aldeen DAA, Wooldridge KG, Turner DPJ: **The moonlighting protein fructose-1, 6-bisphosphate aldolase of *Neisseria meningitidis*: surface localization and role in host cell adhesion.** *Mol Microbiol* 2010, **76:**605-615.

64. Furuya H, Ikeda R: **Interaction of triosephosphate isomerase from the cell surface of *Staphylococcus aureus* and *alpha*-(1 -> 3)-mannooligosaccharides derived from glucuronoxylomannan of *Cryptococcus neoformans*.** *Microbiology - SGM* 2009, **155:**2707-2713.

65. Ikeda R, Saito F, Matsuo M, Kurokawa K, Sekimizu K, Yamaguchi M, Kawamoto S: **Contribution of the mannan backbone of cryptococcal glucuronoxylomannan and a glycolytic enzyme of *Staphylococcus aureus* to contact-mediated killing of *Cryptococcus neofonnans*.** *J Bact* 2007, **189:**4815-4826.

66. Yu NY, Yamauchi T, Yang HF, Chen YL, Gutierrez-Osuna R: **Feature Selection for Inductive Generalization.** *Cognitive Science* 2010, **34:**1574-1593.

67. Kinnby B, Booth NA, Svensaeter G: **Plasminogen binding by oral *streptococci* from dental plaque and inflammatory lesions.** *Microbiology - SGM* 2008, **154:**924-931.

68. Burnham CAD, Shokoples SE, Tyrrell GJ: **Phosphoglycerate kinase inhibits epithelial cell invasion by group B *streptococci*.** *Microb Pathog* 2005, **38:**189-200.

69. Banerjee S, Nandyala AK, Raviprasad P, Ahmed N, Hasnain SE: **Iron-dependent RNA-binding activity of *Mycobacterium tuberculosis* aconitase.** *J Bact* 2007, **189:**4046-4052.

70. La Carbona S, Sauvageot N, Giard JC, Benachour A, Posteraro B, Auffray Y, Sanguinetti M, Hartke A: **Comparative study of the physiological roles of three peroxidases (NADH peroxidase, Alkyl hydroperoxide reductase and Thiol peroxidase) in oxidative stress response, survival inside macrophages and virulence of *Enterococcus faecalis*.** *Mol Microbiol* 2007, **66:**1148-1163.

71. Hu YM, Coates ARM: **Acute and persistent *Mycobacterium tuberculosis* infections depend on the thiol peroxidase TPX.** *Plos One* 2009, **4**.
