# Supplementary material for: The Aeromonas salmonicida subsp. salmonicida exoproteome: global analysis, moonlighting proteins and putative antigens for vaccination against furunculosis
Source: Proteome Sci. 2013 Oct 15;11:44. doi: 10.1186/1477-5956-11-44 (PMC3826670; doi:10.1186/1477-5956-11-44)
Supplement: Additional file 9 — Table.A. salmonicida proteins associated to the outer membrane identified by Ebanks and collaborators [19] and detected in our SNs. The Table shows proteins associated to the A. salmonicida OM identified by Ebanks and collaborators and detected in our SNs and pellets. In grey are indicated proteins with moonlighting activity. [file 1477-5956-11-44-S9.doc]

According to Ebanks,R.O.; Goguen,M.; McKinnon,S.; Pinto,D.M.; Ross,N.W. Identification of the major outer membrane proteins of *Aeromonas salmonicida*. Dis Aquat Organ. 2005 Dec 30;68(1):29-38.

| **Accession Number** | **Protein name of best hit** | **Organism** | **A449 Locus** | **Gene name** |
| --- | --- | --- | --- | --- |
| **OMPs identified in *A. salmonicida* SNs/pellets by our MS analysis** | | | | |
| AAN83357 | Vitamin B12 receptor precursor | *Escherichia coli* CFT073 | ASA_0076 | BtuB |
| AAO09524 | Thiol-disulfide isomerase | *Vibrio vulnificus* | ASA_0109 | DsbA |
| NP_671279 | Protein chain elongation factor EF-Tu | *Yersinia pestis* KIM | ASA_0275 ou ASA_0293 | TufB ou TufA |
| NP_462350 | Protein chain elongation factor EF-G | *Salmonella typhimurium* LT2 | ASA_0292 | fusA |
| NP_668107 | Aconitate hydrase B | *Yersinia pestis* KIM | ASA_0427 | AcnB |
| AAO11460 | Glyceraldehyde-3-phosphate dehydrogenase | *Vibrio vulnificus* | ASA_0759 | Gap |
| AAN54278 | Polyribonucleotide nucleotidyltransferase | *Shewanella oneidensis* MR-1 | ASA_1014 | Pnp |
| CAA63036 | Major outer membrane protein OmpAI | *Aeromonas salmonicida* | ASA_1267 | OmpAI |
| BAA94579 | Iron-cofactored superoxide dismutase | *Aeromonas hydrophila* | ASA_1375 | SodB |
| A39351 | Tetragonal surface virulence array protein | *Aeromonas salmonicida* | ASA_1438 | VapA |
| AAD56398 | Outer membrane protein porin II | *Aeromonas hydrophila* | ASA_1544 | OmpK40 |
| NP_670081 | 30S ribosomal subunit protein S1 | *Yersinia pestis* KIM | ASA_1768 | RpsA |
| BAC59179 | Trigger factor | *Vibrio parahaemolyticus* | ASA_1888 | Tig |
| NP_744587 | Alkyl hydroperoxide reductase, C subunit | *Pseudomonas putida* KT2440 | ASA_2627 | AhpC |
| XP_306658 | Maltose-binding periplasmic protein | *Anopheles gambiae* str. PEST | ASA_2691 | MalE |
| NP_803897 | DnaK heat shock protein 70 | *Salmonella enterica* | ASA_2996 | DnaK |
| CAD29802 | Histone-like nucleoid structuring protein | *Aeromonas hydrophila* | ASA_3010 | Hns |
| NP_670435 | Elongation factor Ts | *Yersinia pestis* KIM | ASA_3159 | Tsf |
| AAN28926 | Enolase | *Aeromonas hydrophila* | ASA_3475 | Eno |
| NP_760438 | 3-phosphoglycerate kinase | *Vibrio vulnificus* CMCP6 | ASA_3505 | Pgk |
| AAN82874 | 2,3-bisphosphoglycerate-independent phosphoglycerate mutase | *Escherichia coli* CFT073 | ASA_4104 | GpmI |
| AAN57375 | Virulence regulator BipA | *Shewanella oneidensis* MR-1 | ASA_4119 | TypA |
| AAC64133 | Phospholipase A1 | *Aeromonas hydrophila* | ASA_4288 | Pla1 |
| **OMPs identified only in *A. salmonicida* pellets by our MS analysis** | | | | |
| AAF93256 | Ferritin | *Vibrio cholerae* | ASA_0049 | Ftn |
| JC6558 | Outer membrane protein A precursor | *Klebsiella pneumoniae* | ASA_0124 | OmpAIV |
| AAN83771 | Carbamate kinase | *Escherichia coli* CFT073 | ASA_0223 | ArcC |
| BAC60927 | ABC transporter, ATP-binding protein | *Vibrio parahaemolyticus* | ASA_0309 | ABC transporter, ATP-binding protein |
| NP_312054 | Phosphoribosylpyrophosphate synthetase | *Yersinia pestis* KIM | ASA_0344 | methyltransferase |
| AAN57187 | Cell division protein FtsZ | *Shewanella oneidensis* MR-1 | ASA_0402 | FtsZ |
| CAA25062 | Outer membrane protein A | *Enterobacter aerogenes* | ASA_0507 | OmpA |
| XP_306643 | Phosphate transport regulator protein | *Anopheles gambiae* str. PEST | ASA_0518 | Phosphate transport regulator protein |
| AAN55775 | Peptidoglycan-associated lipoprotein | *Shewanella oneidensis* MR-1 | ASA_0746 | Pal |
| BAC58431 | Hypothetical TonB-dependent receptor | *Vibrio parahaemolyticus* | ASA_0880 | TonB-dependent receptor |
| XP_306618 | Riboflavin synthase, β-chain | *Anopheles gambiae* str. PEST | ASA_0982 | RibH |
| ZP_00015887 | Hypothetical protein | *Rhodospirillum rubrum* | ASA_1101 | Hypothetical protein |
| CAA63037 | Major outer membrane protein OmpAII | *Aeromonas salmonicida* | ASA_1266 | OmpAII |
| NP_798627 | Polar flagellar motor switch protein FliG | *Vibrio parahaemolyticus* | ASA_1338 | FliG |
| AAN57289 | Agglutination protein | *Shewanella oneidensis* MR-1 | ASA_1671 | Type I secretion outer membrane protein, TolC |
| AAN81146 | Colicin I receptor precursor | *Escherichia coli* CFT073 | ASA_1850 | FstC |
| AAO07170 | Fructose-specific phosphotranferase system | *Vibrio vulnificus* | ASA_1941 | FruA |
| Q9KSX1 | Histidinol-phosphatase | *Vibrio cholerae* | ASA_2104 | HisB |
| AAN81339 | Long chain fatty acid transport protien | *Escherichia coli* K12 | ASA_2156 ou ASA_2157 | FadL1 ou FadL2 |
| CAC07184 | Exo-1,4-beta-glucosidase | *Prevotella albensis* | ASA_2542 | beta-glucosidase |
| AAF95396 | Surface antigen | *Vibrio cholerae* | ASA_3152 | Surface antigen |
| S37779 | Maltose-inducible porin precursor | *Aeromonas salmonicida* | ASA_3168 | LamB |
| XP_306378 | Outer membrane protein OmpK | *Anopheles gambiae* str. PEST | ASA_3206 | OmpK |
| B82202 | Thermostable carboxypeptidase | *Vibrio cholerae* | ASA_3252 | thermostable carboxypeptidase 1 |
| O08437 | FKBP-type peptidyl-prolyl isomerase | *Aeromonas hydrophila* | ASA_3296 | FkpA |
| AAN56622 | Organic solvent tolerance protein | *Shewanella oneidensis* MR-1 | ASA_3347 | Imp |
| AAN53683 | HlfK protein | *Shewanella oneidensis* MR-1 | ASA_3367 | HflK |
| NP_414739 | Putative lipoprotein | *Escherichia coli K12* | ASA_3380 | MetQ |
| D82129 | Hypothetical protein VC2002 | *Vibrio cholerae* | ASA_3388 | Hypothetical protein |
| AAC09226 | Chaperonin GroEL | *Aeromonas salmonicida* | ASA_3431 | GroEL |
| P37592 | Outer membrane porin protein OmpD | *Salmonella typhimurium* LT2 | ASA_3437 | OmpC |
| AAO09234 | Sulfate adenylate transferase subunit | *Vibrio vulnificus* CMCP6 | ASA_3522 | CysN |
| CAC89297 | Purine nucleoside phosphorylase | *Yersinia pestis* | ASA_3652 | DeoD |
| CAA56668 | ExeD protein | *Aeromonas salmonicida* | ASA_3775 | ExeD |
| XP_306408 | 3’-phosphoadenosine 5’-phosphosulfate | *Anopheles gambiae* str. PEST | ASA_3790 | CysQ |
| AAO09596 | Enzyme of heme biosynthesis | *Vibrio vulnificus* | ASA_3877 | HemX |
| BAC60954 | Rod shape-determining protein MreB | *Vibrio parahaemolyticus* | ASA_3937 | MreB |
| BAA11840 | DNA-directed RNA polymerase α-subunit | *Shewanella oneidensis* MR-1 | ASA_4062 | RpoA |
| XP_306258 | Gluconate transcriptional repressor | *Anopheles gambiae* str. PEST | ASA_4107 | PurR |
| NP_667684 | Keto-acid reductoisomerase | *Yersinia pestis* KIM | ASA_4236 | IlvC |
| XP_306428 | Acetoacetyl-CoA synthetase | *Anopheles gambiae* str. PEST | ASA_4249 | AcsA |
| D64071 | H+-transporting 2-sector ATPase | *Haemophilus influenzae* | ASA_4350 | AtpD |
| NP_462766 | ATP synthase α-subunit | *Salmonella typhimurium* LT2 | ASA_4352 | AtpA |
| **OMPs not identified in our MS analysis** | | | | |
| BAC51234 | Putative phenylacetate-CoA ligase | *Bradyrhizobium japonicum* USDA | ASA_1253 | anaerobic phenylacetate CoA ligase |
| AAO70632 | Conserved hypothetical protein | *Salmonella enterica* | ASA_1276 | YgiQ-like |
| BAC60489 | Conserved hypothetical protein | *Vibrio parahaemolyticus* | ASA_1362 | / |
| AAF95082 | GGDEF family protein | *Vibrio cholerae* | ASA_2497 | GGDEF/EAL domain protein |
| A39442 | Conjugative transfer protein trbC | *Escherichia coli* | ASA_P5G034 | TrbC |
