# Supplementary material for: The Aeromonas salmonicida subsp. salmonicida exoproteome: global analysis, moonlighting proteins and putative antigens for vaccination against furunculosis
Source: Proteome Sci. 2013 Oct 15;11:44. doi: 10.1186/1477-5956-11-44 (PMC3826670; doi:10.1186/1477-5956-11-44)
Supplement: Additional file 11 — Table.A. salmonicida proteins identified in SNs or associated to the OM that have antigenic homologues in other bacteria and constitute candidates for subunit vaccine. [file 1477-5956-11-44-S11.doc]

| **Locus** | **Description** |  | **Homologues** | **Host response and antigenic protection** | **Reference** |
| --- | --- | --- | --- | --- | --- |
| **Supernatant** | | | | | |
| ASA_P5G009 | AopH | T3SS effector | *Yersinia* YopH | Anti-inflammatory  Immune suppressor  Non-protective  Immunogen | [1-4] |
| ASA_4266 | AexT | T3SS effector | *Yersinia* YopE | Anti-inflammatory  Protective antigen | [5-7] |
|  |  |  |  | Immunogen but non-protective | [3,4,8] |
| pAsal1_03 | AopP | T3SS effector | *Yersinia* YopJ | Anti-inflammatory  Immune suppressor | [2,5] |
| ASA_P5G098 | AopO | T3SS effector | *Yersinia* YpkA | Putative immune suppressor  Immunogen but non-protective | [2,4] |
| ASA_P5G075 | AopN | T3SS effector | *Bordetella* BopN | Immune suppressor | [9] |
|  |  |  | *Pseudomonas syringae* HrpJ | Immune suppressor | [10] |
|  |  |  | *Yersinia* YopN | Antigenic but non-protective | [4,8] |
|  |  |  | *Chlamydia* CopN | Protective antigen | [11] |
| ASA_P5G065 | AopB | T3SS translocon | *Yersinia* YopB | Pro-inflammatory  Immune suppressor  Protective antigen | [5,12,13] |
|  |  |  | *Salmonella enterica* SipB | Inflammation suppression | [14] |
| ASA_P5G064 | AopD | T3SS translocon | *Yersinia* YopD | Immunogen  Protective antigen | [3,4,13] |
|  |  |  | *Salmonella enterica* serovar *enteritidis* SipD | Partially protective immunogen | [15] |
|  |  |  | *Edwardsiella tarda* EseD | Protective antigen | [16] |
| ASA_P5G067 | AcrV | T3SS translocon | *Yersinia* LcrV | Anti-inflammatory  Immune suppressor  Protective antigen | [12,13,17-20] |
|  |  |  | *Pseudomonas aeruginosa* PcrV | Protective antigen | [21] |
| ASA_P5G054 | AscF | T3SS needle | *Yersinia* YscF | Partial protective antigen | [22,23] |
| ASA_P5G078 | AscP | T3SS needle | *Yersinia* YscP | Immunogenic | [24] |
| ASA_0292 | EF-G | Elongation factor G | *Neisseria meningitidis* | Immunogenic | [25] |
|  |  |  | *Streptococcus suis* | Immunogenic | [26] |
|  |  |  | *Paenibacillus larvae* | Immunogenic | [27] |
|  |  |  | *Riemerella anatipestifer* | Immunogenic | [28,29] |
|  |  |  | *Francisella tularensis* | Immunogenic | [30] |
|  |  |  | *Bacillus anthracis, thuringiensis* | Immunogenic | [31] |
|  |  |  | *Lactococcus garvieae* | Immunogenic | [32] |
|  |  |  | *Flavobacterium columnare* | Immunogenic | [33] |
|  |  |  | *Chlamydia trachomatis* | Immunogenic | [34] |
| ASA_0275  ASA_0293 | EF-Tu | Elongation factor Tu | *Neisseria meningitidis* | Immunogenic | [25] |
|  |  |  | *Chlamydia trachomatis* | Immunogenic | [35] |
|  |  |  | *Francisella tularensis* | Immunogenic | [30,36] |
|  |  |  | *Bordetella pertussis* | Immunogenic | [37] |
|  |  |  | *Burkholderia thailandensis* | Immunogenic and partially protective | [38] |
|  |  |  | *Burkholderia cepacia* | Immunogenic | [39] |
|  |  |  | *Mycobacterium immunogenum* | Immunogenic | [40] |
|  |  |  | *Streptococcus suis* | Immunogenic | [26] |
|  |  |  | *Mycobacterium bovis* | Antigenic | [41] |
|  |  |  | *Actinobacillus pleuropneumoniae* | Immunogenic | [42] |
|  |  |  | *Bacillus anthracis*, *cereus*, *thuringiensis* | Immunogenic | [31] |
|  |  |  | *Lactococcus garvieae* | Immunogenic | [32] |
|  |  |  | *Flavobacterium columnare* | Immunogenic | [33] |
|  |  |  | *Chlamydia trachomatis* | Immunogenic | [34] |
|  |  |  | *Chlamydia pneumoniae* | Immunogenic | [43] |
| ASA_2996 | DnaK | Chaperone protein | *Neisseria meningitidis* | Immunogenic | [25] |
|  |  |  | *Salmonella enterica* serovar *Typhi* | Protective antigen | [44] |
|  |  |  | *Brucella abortus* | Immunogenic and partially protective | [45] |
|  |  |  | *Brucella ovis* | Immunogenic | [46] |
|  |  |  | *Chlamydia trachomatis* | Immunogenic | [35] |
|  |  |  | *Francisella tularensis* | Immunogenic | [36] |
|  |  |  | *Burkholderia* | Immunogenic and partially protective | [38] |
|  |  |  | *Streptococcus suis* | Immunogenic | [26] |
|  |  |  | *Edwardsiella tarda* | Immunigenic and protective | [47] |
|  |  |  | *Riemerella anatipestifer* | Immunogenic | [28] |
|  |  |  | *Streptococcus pneumoniae* | Non-protective immunogen | [48] |
|  |  |  | *Bacillus anthracis* | Non-protective immunogen | [49] |
|  |  |  | *Flavobacterium psychrophilum* | Non-protective immunogen | [50] |
|  |  |  | *Mycobacterium bovis* | Antigenic | [41] |
|  |  |  | *Mycoplasma haemofelis* | Immunogenic | [51] |
|  |  |  | *Flavobacterium columnare* | Immunogenic | [33] |
|  |  |  | *Chlamydia trachomatis* | Immunogenic | [34] |
|  |  |  | *Chlamydia pneumoniae* | Immunogenic | [43] |
| ASA_3159 | EF-Ts | Elongation factor Ts | *Neisseria meningitidis* | Immunogenic | [25] |
|  |  |  | *Francisella tularensis* | Immunogenic | [36] |
|  |  |  | *Mycoplasma haemofelis* | Immunogenic | [51] |
|  |  |  | *Bordetella pertussis* | Immunogenic | [37] |
| ASA_1086 | EF-P | Elongation factor P | *Chlamydia trachomatis* | Immunogenic | [34] |
| ASA_1247  ASA_1614  ASA_3203 | PpiA, B and C | FKBP-type peptidyl-prolyl cis-trans isomerase | *Chlamydia muridarum* | Immunodominant protective antigen | [52] |
|  |  |  | *Neisseria meningitidis* | Protective immunogen | [53] |
|  |  |  | *Actinobacillus pleuropneumoniae* | Immunogenic | [42] |
| ASA_1888 | Tig | Tigger factor | *Actinobacillus pleuropneumoniae* | Immunogenic | [42] |
|  |  |  | *Flavobacterium columnare* | Immunogenic | [33] |
| ASA_1826 | HtpG | Chaperone protein | *Borrelia burgdorferi* | Immunogenic | [54] |
|  |  |  | *Francisella tularensis* | Immunogenic | [36] |
|  |  |  | *Burkholderia* | Immunogenic and partially protective | [38] |
|  |  |  | *Porphyromonas gingivalis* | Protective antigen | [55] |
| ASA_1768 | RpsA | 30S ribosomal protein S1 | *Chlamydia trachomatis* | Immunogenic | [35] |
|  |  |  | *Neisseria meningitidis* | Immunogenic | [25] |
|  |  |  | *Burkholderia* | Immunogenic and partially protective | [38] |
|  |  |  | *Francisella tularensis* | Immunogenic | [30] |
| ASA_2627 | AhpC | Alkyl hydroperoxide reductase subunit C | *Burkholderia* | Immunogenic and partially protective | [38] |
|  |  |  | *Campylobacter jejuni* | Antigenic | [56] |
|  |  |  | *Helicobacter pylori* | Protective antigen | [57] |
|  |  |  | *Bacillus anthracis*, *cereus*, *thuringiensis* | Immunogenic | [31] |
|  |  |  | *Chlamydia trachomatis* | Immunogenic | [34] |
| ASA_1375 | SodB | Superoxide dismutase | *Bordetella pertussis* | Immunogenic | [58] |
| ASA_0402 | FtsZ | Cell division protein | *Neisseria meningitidis* | Immunogenic | [25] |
|  |  |  | *Borrelia burgdorferi* | Immunogenic | [54] |
|  |  |  | *Bartonella bacilliformis* | Antigenic | [59] |
|  |  |  | *Rickettsia parkeri* | Immunogenic | [60] |
|  |  |  | *Streptococcus iniae* | Immunogenic | [61] |
| ASA_3059 | CysK | Cysteine synthase | *Neisseria meningitidis* | Immunogenic | [25] |
|  |  |  | *Brucella abortus* | Protective antigen | [62] |
| ASA_1827 | Adk | Adenylate kinase | *Neisseria meningitidis* | Immunogenic | [25] |
| ASA_0427 | AcnB | Aconitate hydratase | *Neisseria meningitidis* | Immunogenic | [25] |
| ASA_1202 | Tkt1 | Tranketolase 1 | *Francisella tularensis* | Immunogenic | [36] |
|  |  |  | *Bacillus anthracis* | Highly immunogenic and putatively protective | [63] |
| ASA_0759 | GAPDH | Glyceraldehyde-3-phosphoglycerate dehydrogenase | *Aeromonas hydrophila* | Protective antigen | [64] |
|  |  |  | *Edwardsiella tarda* | Protective antigen | [65] |
|  |  |  | *Francisella tularensis* | Immunogenic | [36] |
|  |  |  | *Streptococcus pneumoniae* | Protective antigen | [48] |
|  |  |  | *Streptococcus iniae* | Immunogenic | [61] |
|  |  |  | *Actinobacillus pleuropneumoniae* | Immunogenic | [42] |
|  |  |  | *Clostridium perfringens* | Protective antigen | [66] |
|  |  |  | *Bacillus anthracis*, *cereus*, *thuringiensis* | Immunogenic | [31] |
|  |  |  | *Bordetella pertussis* | Immunogenic | [37] |
| ASA_3475 | Eno | Enolase | *Paenibacillus larvae* | Immunogenic | [27] |
|  |  |  | *Bacillus anthracis* | Highly immunogenic and putatively protective | [63] |
|  |  |  | *Bacillus anthracis*, *cereus*, *thuringiensis* | Immunogenic | [31] |
|  |  |  | *Streptococcus pneumoniae* | Immunogenic | [48] |
|  |  |  | *Streptococcus iniae* | Immunogenic | [61] |
|  |  |  | *Riemerella anatipestifer* | Immunogenic | [28] |
| ASA_3504 | FbaA | Fructose-bisphosphate aldolase class II | *Streptococcus pneumoniae* | Protective antigen | [48] |
|  |  |  | *Streptococcus iniae* | Immunogenic | [61] |
|  |  |  | *Clostridium perfringens* | Protective antigen | [66,67] |
|  |  |  | *Francisella tularensis* | Immunogenic | [30] |
|  |  |  | *Edwardsiella ictaluri* | Immunogenic | [68] |
|  |  |  | *Flavobacterium columnare* | Immunogenic | [33] |
| ASA_0659 | Mdh | Malate dehydrogenase | *Francisella tularensis* | Immunogenic | [36] |
|  |  |  | *Actinobacillus pleuropneumoniae* | Immunogenic | [42] |
|  |  |  | *Brucella abortus* | Protective antigen | [69] |
|  |  |  | *Brucella ovis* | Immunogenic | [46] |
| ASA_3505 | Pgk | Phosphoglycerate kinase | *Streptococcus pneumoniae* | Immunogenic | [48] |
|  |  |  | *Streptococcus agalactiae* | Protective antigen | [70] |
|  |  |  | *Edwardsiella ictaluri* | Immunogenic | [68] |
|  |  |  | *Mycoplasma haemofelis* | Immunogenic | [51] |
| ASA_3402 | Pta | Phosphate acetyltransferase | *Actinobacillus pleuropneumoniae* | Immunogenic | [42] |
| ASA_1014 | Pnp | Polyribonucleotide nucleotidyltransferase | *Chlamydia trachomatis* | Immunogenic | [34] |
| ASA_2540 | Ahe2 | Serine protease | *Aeromonas salmonicida* | Strong immune suppressor | [71] |
| ASA_0509 | SatA | Glycero-phospholipid-cholesterol acyltransferase | *Aeromonas salmonicida* | Immunogenic, mild stimulation | [71] |
| ASA_1438 | VapA | Tetragonal surface virulence array protein | *Aeromonas salmonicida* | Immunogenic and partially protective | [72-74] |
|  |  |  | *Aeromonas salmonicida* atypical | Immunogenic but non-protective | [75] |
|  |  |  | *Aeromonas salmonicida* | Protective antigen against homologous *A. salmonicida* | [76] |
| ASA_3906 | AerA | Aerolysin A | *Aeromonas hydrophila* | D1 and D4 regions immunogenic and protective by injection or orally when expressed in Lactococcus lactis | [77-79] |
| ASA_2854 | AerB | Aerolysin B | *Aeromonas hydrophila* | Immunogenic | [78] |
| ASA_0826 | Asx | RTX protein | *Escherichia coli* (UPEC) | Antigenic | [80] |
| ASA_2206 | NucH | Nuclease | *Aeromonas hydrophila* | Immunogenic | [78] |
| ASA_3321 | TagA | Metalloprotease/  mucinase ToxR-regulated lipoprotein | *Aeromonas hydrophila* | Immunogenic | [78] |
|  |  |  | *Escherichia coli* (STEC) | Immunogenic | [81] |
| ASA_0873 | CdxA | Chitinase | *Francisella tularensis* | Immunogenic | [36] |
| ASA_0604 | ChiY | Chitin-binding protein | *Aeromonas hydrophila* | Immunogenic | [78] |
| ASA_1661 |  | Extracellular protease | *Aeromonas hydrophila* | Protective antigen | [82] |
|  |  |  | *Paenibacillus larvae* | Immunogenic | [27] |
| ASA_0849 | PrtV | immune inhibitor A metalloprotease | *Bacillus anthracis* | Immunogenic | [83] |
| ASA_1889 | ClpP | ATP-dependent Clp protease | *Brucella ovis* | Immunogenic | [46] |
|  |  |  | *Streptococcus pneumoniae* | Protective antigen | [84,85] |
|  |  |  | *Bacillus anthracis*, *cereus*, *thuringiensis* | Immunogenic | [31] |
|  |  |  | *Chlamydia trachomatis* | Immunogenic | [34] |
| ASA_0656 | ArtI | ABC-type arginine transporter, periplasmic binding protein | *Bordetella pertussis* | Immunogenic | [58] |
|  |  |  | *Chlamydia trachomatis* | Immunogenic | [34] |
| ASA_0975 | GlyA | Serine hydroxymethyltransferase | *Streptococcus iniae* | Immunogenic | [61] |
| ASA_3982 | TAXI | TRAP transporter solute receptor, TAXI (TRAP-associated extracytoplasmic immunogenic) family | *Brucella abortus* | Immunogenic | [86] |
| ASA_2355 | SucC | Succinyl-CoA synthetase subunit beta | *Bordetella pertussis* | Immunogenic | [37] |
| ASA_0330 | DegQ | Serine protease | *Chlamydia trachomatis* | Immunogenic | [34] |
| ASA_1891 | Lon | Lon protease | *Chlamydia trachomatis* | Immunogenic | [34] |
| ASA_4001 | CspA | Major cold shock protein | *Edwardsiella Tarda* | Immunogenic | [87] |
| ASA_2359 | SdhA | Succinate dehydrogenase flavoprotein subunit | *Bordetella pertussis* | Immunogenic | [37] |
| ASA_2942 | Icd | Isocitrate dehydrogenase | *Bordetella pertussis* | Immunogenic | [37] |
| ASA_3380 | MetQ | Lipoprotein | *Bordetella pertussis* | Immunogenic | [37] |
| ASA_1511 | Upp | Uracil phosphoribosyltransferase | *Chlamydia trachomatis* | Immunogenic | [34] |
| ASA_1768 | RpsA | 30S ribosomal protein S1 | *Flavobacterium columnare* | Immunogenic | [33] |
|  |  |  | *Neisseria meningitidis* | Immunogenic | [25] |
|  |  |  | *Chlamydia trachomatis* | Immunogenic | [34] |
| ASA_0707 | RpsF | 30S ribosomal protein S6 | *Edwardsiella Tarda* | Immunogenic | [87] |
| ASA_4070 | RpsE | 30S ribosomal protein S5 | *Chlamydia trachomatis* | Immunogenic | [34] |
| ASA_4078 | RpsH | 30S ribosomal protein S8 | *Chlamydia trachomatis* | Immunogenic | [34] |
| ASA_0709 | RplI | 50S ribosomal protein L9 | *Edwardsiella Tarda* | Immunogenic | [87] |
| ASA_0281 | RplJ | 50S ribosomal protein L10 | *Flavobacterium psychrophilum* | Protective antigen | [88] |
| Ribosomal proteins |  |  | *Shigella flexneri* | Protective antigens | [89] |
|  |  |  | *Salmonella typhimurium* | Protective antigens | [90] |
|  |  |  | *Klebsiella pneumoniae* | Protective antigens | [91] |
| **Outer Membrane proteins** | | | | | |
| ASA_P5G050 | AscJ | T3SS MS ring, OM secretin ring | *Yersinia* YscC | Immunogenic | [24] |
|  |  |  | *Chlamydia trachomatis* | Immunogenic | [34] |
| ASA_P5G048 | AscL | T3SS Interactors of ATPase/C ring, ATPase | *Chlamydia trachomatis* | Immunogenic | [34] |
| ASA_P5G057 | AscC | T3SS OM ring | *Yersinia* YscC | Immunogenic | [92] |
| ASA_P5G077 | AscO | T3SS chaperone | *Yersinia* YscO | Immunogenic | [24] |
| ASA_P5G074 | Acr1 | T3SS chaperone | *Yersinia* TyeA | Immunogenic | [24] |
| ASA_3431 | GroEL | Chaperonin (HSP60) | *Bordetella pertussis* | Immunogenic | [37] |
|  |  |  | *Bacillus anthracis* | Protective antigen | [49] |
|  |  |  | *Salmonella enterica* serovar *typhi* | Protective antigen | [93,94] |
|  |  |  | *Helicobacter pylori* | Protective antigen | [95] |
|  |  |  | *Ehrlichia muris* | Protective antigen | [96] |
|  |  |  | *Brucella abortus* | Non-protective antigen | [97] |
|  |  |  | *Porphyromonas gingivalis* | Immunogenic | [98] |
|  |  |  | *Chlamydia trachomatis* | Immunogenic | [34] |
|  |  |  | *Chlamydia pneumoniae* | Immunogenic | [43] |
|  |  |  | *Flavobacterium psychrophilum* | Non-protective immunogen | [50] |
|  |  |  | *Riemerella anatipestifer* | Immunogenic | [29] |
| ASA_0518 |  | Phosphate transport regulator | *Chlamydia trachomatis* | Immunogenic | [34] |
| ASA_3152 | BamA | Outer membrane protein assembly factor BamA, Surface antigen | *Chlamydia trachomatis* | Immunogenic | [34] |
| ASA_1267 | OmpAI | Outer membrane protein AI | *Aeromonas salmonicida* | Partially protective antigen | [99] |
|  |  |  | *Pasteurella multocida* | Major protective antigen | [100] |
|  |  |  | *Yersinia pestis* | Protective antigen | [101] |
|  |  |  | *Mannheimia haemolytica* | Major immunogen | [102] |
|  |  |  | *Edwardsiella Tarda* | Immunogenic | [87] |
| ASA_1544 | OmpK40 | Outer membrane protein K40 | *Aeromonas hydrophila* (Aha1, Omp38) | Protective antigen  Cross protection | [103-107] |
|  |  |  | *Pasteurella multocida* | Major protective antigen | [100] |
|  |  |  | *Vibrio harveyi* | Major protective antigen | [108] |
|  |  |  | *Vibrio parahaemolyticus* | Major protective antigen | [109] |
| ASA_P5G061 | ExsB | T3SS outer membrane ring stabilisator | *Yersinia pestis* (YscW) | Immune suppressor | [110] |
| ASA_0746 | Pal | Peptidoglycan-associated lipoprotein | *Aliivibrio salmonicida* | Immunogenic | [111] |
|  |  |  | *Aggregatibacter actinomycetemcomitans* | Highly immunogenic | [112] |
|  |  |  | *Bordetella pertussis* | Immunodominant antigen | [113] |
|  |  |  | *Campylobacter jejuni* | Immunogenic | [114] |
|  |  |  | *Legionella pneumophila* | Immunogenic | [115] |
| ASA_0880 |  | TonB-dependent receptor | *Riemerella anatipestifer* | Immunogenic | [29] |
| ASA_3168  (ASA_2388) | LamB2  (LamB) | Maltoporin (Maltose-inducible porin) | *Aeromonas hydrophila* | Protective antigen | [116] |
|  |  |  | *Salmonella paratyphi* A | Protective antigen | [117] |
| ASA_0523  (ASA_1671) | TolC | Type I secretion outer membrane protein | *Salmonella paratyphi* A | Protective antigen | [117] |
| ASA_3437 | OmpC | Outer membrane protein C | *Salmonella paratyphi* A | Non-protective antigen | [117] |
|  |  |  | *Vibrio parahaemolyticus* | Immunogenic | [109] |
| ASA_2156  (ASA_2157) | FadL | Long-chain fatty acid transport protein | *Salmonella paratyphi* A | Protective antigen | [117] |
| ASA_0076 | BtuB | Vitamin B12 transporter BtuB | *Salmonella paratyphi* A | Partially protective antigen | [117] |
| ASA_3347 | Imp | Organic solvent tolerance protein | *Salmonella paratyphi* A | Non-protective antigen | [117] |
| ASA_3206 | OmpK | Outer membrane protein OmpK | *Vibrio parahaemolyticus* | Immunogenic | [108,109] |
| ASA_4062 | RpoA | DNA-directed RNA polymerase subunit alpha | *Chlamydia trachomatis* | Immunogenic | [34] |
|  |  |  | *Chlamydia pneumoniae* | Immunogenic | [43] |

**References:**

1. Blanco HM, Lacoste MG, Elicabe RJ, Di Genaro MS: **IgA response by oral infection with an attenuated *Yersinia enterocolitica* mutant: implications for its use as oral carrier vaccine.** *Vaccine* 2008, **26:**6497-6502.

2. Maia JM, Monnazzi LG, Medeiros BM: **Role of *Yersinia pseudotuberculosis* outer proteins (Yops) in murine humoral immune response.** *Folia Microbiol* 2009, **54:**239-245.

3. Benner GE, Andrews GP, Byrne WR, Strachan SD, Sample AK, Heath DG, Friedlander AM: **Immune response to *Yersinia* outer proteins and other *Yersinia pestis* antigens after experimental plague infection in mice.** *Infect Immun* 1999, **67:**1922-1928.

4. Andrews GP, Strachan ST, Benner GE, Sample AK, Anderson GW, Adamovicz JJ, Welkos SL, Pullen JK, Friedlander AM: **Protective efficacy of recombinant *Yersinia* outer proteins against bubonic plague caused by encapsulated and nonencapsulated *Yersinia pestis*.** *Infect Immun* 1999, **67:**1533-1537.

5. Viboud GI, So SSK, Ryndak MB, Bliska JB: **Proinflammatory signalling stimulated by the type III translocation factor YopB is counteracted by multiple effectors in epithelial cells infected with *Yersinia pseudotuberculosis*.** *Mol Microbiol* 2003, **47:**1305-1315.

6. Zhang Y, Mena P, Romanov G, Lin JS, Smiley ST, Bliska JB: **A protective epitope in type III effector YopE is a major CD8 T cell antigen during primary infection with *Yersinia pseudotuberculosis*.** *Infect Immun* 2012, **80:**206-214.

7. Lin JS, Szaba FM, Kummer LW, Chromy BA, Smiley ST: ***Yersinia pestis* YopE contains a dominant CD8 T cell epitope that confers protection in a mouse model of pneumonic plague.** *J Immunol* 2011, **187:**897-904.

8. Leary SEC, Griffin KF, Galyov EE, Hewer J, Williamson ED, Holmstrom A, Forsberg A, Titball RW: ***Yersinia* outer proteins (YOPS) E, K and N are antigenic but non-protective compared to V antigen, in a murine model of bubonic plague.** *Microb Pathogenesis* 1999, **26:**159-169.

9. Nagamatsu K, Kuwae A, Konaka T, Nagai S, Yoshida S, Eguchi M, Watanabe M, Mimuro H, Koyasu S, Abe A: ***Bordetella* evades the host immune system by inducing IL-10 through a type III effector, BopN.** *J Exp Med* 2009, **206:**3073-3088.

10. Crabill E, Karpisek A, Alfano JR: **The *Pseudomonas syringae* HrpJ protein controls the secretion of type III translocator proteins and has a virulence role inside plant cells.** *Mol Microbiol* 2012, **85:**225-238.

11. Tammiruusu A, Penttila T, Lahesmaa R, Sarvas M, Puolakkainen M, Vuola JM: **Intranasal administration of chlamydial outer protein N (CopN) induces protection against pulmonary *Chlamydia pneumoniae* infection in a mouse model.** *Vaccine* 2007, **25:**283-290.

12. Sodhi A, Sharma RK, Batra HV, Tuteja U: **Mechanism of rLcrV and rYopB mediated Immunosuppression in murine peritoneal macrophages.** *Mol Immunol* 2004, **41:**767-774.

13. Ivanov MI, Noel BL, Rampersaud R, Mena P, Benach JL, Bliska JB: **Vaccination of mice with a Yop translocon complex elicits antibodies that are protective against infection with F1(-) *Yersinia pestis*.** *Infect Immun* 2008, **76:**5181-5190.

14. Pavlova B, Volf J, Ondrackova P, Matiasovic J, Stepanova H, Crhanova M, Karasova D, Faldyna M, Rychlik I: **SPI-1-encoded type III secretion system of *Salmonella enterica* is required for the suppression of porcine alveolar macrophage cytokine expression.** *Vet Res* 2011, **42**.

15. Desin TS, Wisner ALS, Lam PKS, Berberov E, Mickael CS, Potter AA, Koster W: **Evaluation of *Salmonella enterica* serovar *enteritidis* pathogenicity island-1 proteins as vaccine candidates against *S. enteritidis* challenge in chickens.** *Vet Microbiol* 2011, **148:**298-307.

16. Wang B, Mo ZL, Xiao P, Li J, Zou YX, Hao B, Li GY: **EseD, a putative T3SS translocon component of *Edwardsiella tarda*, contributes to virulence in fish and is a candidate for vaccine development.** *Mar Biotechnol* 2010, **12:**678-685.

17. Overheim KA, DePaolo RW, Debord KL, Morrin EM, Anderson DM, Green NM, Brubaker RR, Jabri B, Schneewind O: **LcrV plague vaccine with altered immunomodulatory properties.** *Infect Immun* 2005, **73:**5152-5159.

18. Gendrin C, Sarrazin S, Bonnaffe D, Jault JM, Lortat-Jacob H, Dessen A: **Hijacking of the pleiotropic cytokine interferon-gamma by the type-III secretion system of *Yersinia pestis*.** *Plos One* 2010, **5**.

19. Sing A, Rost D, Tvardovskaia N, Roggenkamp A, Wiedemann A, Kirschning CJ, Aepfelbacher M, Heesemann J: ***Yersinia* V-antigen exploits toll-like receptor 2 and CD14 for interleukin 10-mediated immunosuppression.** *J Exp Med* 2002, **196:**1017-1024.

20. Brubaker RR: **Interleukin-10 and inhibition of innate immunity to yersiniae: roles of Yops and LcrV (V antigen).** *Infect Immun* 2003, **71:**3673-3681.

21. Sawa T, Yahr TL, Ohara M, Kurahashi K, Gropper MA, Wiener-Kronish JP, Frank DW: **Active and passive immunization with the *Pseudomonas* V antigen protects against type III intoxication and lung injury.** *Nat Med* 1999, **5:**392-398.

22. Matson JS, Durick KA, Bradley DS, Nilles ML: **Immunization of mice with YscF provides protection from *Yersinia pestis* infections.** *BMC Microbiol* 2005, **5**.

23. Swietnicki W, Powell BS, Goodin J: ***Yersinia pestis* Yop secretion protein F: purification, characterization, and protective efficacy against bubonic plague.** *Protein Expres Purif* 2005, **42:**166-172.

24. Hill J, Underwood CD, Sundberg L, Astrom H, Leary SEC, Forsberg A,Titball RW: **Immunological characterisation of sub-units of the *Yersinia* type III secretion apparatus.** 2003:415-417.

25. Mendum TA, Newcombe J, McNeilly CL, McFadden J: **Towards the immunoproteome of *Neisseria meningitidis*.** *Plos One* 2009, **4**.

26. Wu ZF, Zhang W, Shao J, Wang Y, Lu Y, Lu CP: **Immunoproteomic assay of secreted proteins of *Streptococcus suis* serotype 9 with convalescent sera from pigs.** *Folia Microbiol* 2011, **56:**423-430.

27. Antunez M, Anido M, Evans JD, Zunino P: **Secreted and immunogenic proteins produced by the honeybee bacterial pathogen, *Paenibacillus larvae*.** *Vet Microbiol* 2010, **141:**385-389.

28. Zhai ZP, Cheng LF, Tang F, Lu Y, Shao J, Liu GJ, Bao YL, Chen MM, Shang KX, Fan HJ et al.: **Immunoproteomic identification of 11 novel immunoreactive proteins of *Riemerella anatipestifer* serotype 2.** *FEMS Immunol Med Mic* 2012, **65:**84-95.

29. Hu QH, Ding C, Tu J, Wang XL, Han XA, Duan YB, Yu SQ: **Immunoproteomics analysis of whole cell bacterial proteins of *Riemerella anatipestifer*.** *Vet Microbiol* 2012, **157:**428-438.

30. Twine S, Shen H, Harris G, Chen WX, Sjostedt A, Ryden P, Conlan W: **BALB/c mice, but not C57BL/6 mice immunized with a *Delta* *clpB* mutant of *Francisella tularensis* subspecies *tularensis* are protected against respiratory challenge with wild-type bacteria: Association of protection with post-vaccination and post-challenge immune responses.** *Vaccine* 2012, **30:**3634-3645.

31. DelVecchio VG, Connolly JP, Alefantis TG, Walz A, Quan MA, Patra G, Ashton JM, Whittington JT, Chafin RD, Liang XD et al.: **Proteomic profiling and identification of immunodominant spore antigens of *Bacillus anthracis*, *Bacillus cereus*, and *Bacillus thuringiensis*.** *Appl Environ Microbiol* 2006, **72:**6355-6363.

32. Shin GW, Palaksha KJ, Kim YR, Nho SW, Cho JH, Heo NE, Heo GJ, Park SC, Jung TS: **Immunoproteomic analysis of capsulate and non-capsulate strains of *Lactococcus garvieae*.** *Vet Microbiol* 2007, **119:**205-212.

33. Liu ZX, Liu GY, Li N, Xiao FS, Xie HX, Nie P: **Identification of immunogenic proteins of *Flavobacterium columnare* by two-dimensional electrophoresis immunoblotting with antibacterial sera from grass carp, *Ctenopharyngodon idella* (Valenciennes).** *J Fish Dis* 2012, **35:**255-263.

34. Cruz-Fisher MI, Cheng CM, Sun GF, Pal S, Teng A, Molina DM, Kayala MA, Vigil A, Baldi P, Felgner PL et al.: **Identification of immunodominant antigens by probing a whole *Chlamydia trachomatis* open reading frame proteome microarray using sera from immunized mice.** *Infect Immun* 2011, **79:**246-257.

35. Sanchez-Campillo M, Bini L, Comanducci R, Raggiaschi R, Marzocchi B, Pallini V, Ratti G: **Identification of immunoreactive proteins of *Chlamydia trachomatis* by Western blot analysis of a two-dimensional electrophoresis map with patient sera.** *Electrophoresis* 1999, **20:**2269-2279.

36. Havlasova J, Hernychova L, Brychta M, Hubalek M, Lenco J, Larsson P, Lundqvist M, Forsman M, Krocova Z, Stulik J et al.: **Proteomic analysis of anti-*Franciselia tularensis* LVS antibody response in murine model of tularemia.** *Proteomics* 2005, **5:**2090-2103.

37. Zhu YZ, Cai CS, Zhang W, Guo HX, Zhang JP, Ji YY, Ma GY, Wu JL, Li QT, Lu CP et al.: **Immunoproteomic analysis of human serological antibody responses to vaccination with whole-cell pertussis vaccine (WCV).** *Plos One* 2010, **5**.

38. Nieves W, Heang J, Asakrah S, Bentrup KHZ, Roy CJ, Morici LA: **Immunospecific responses to bacterial elongation factor Tu during *Burkholderia* infection and immunization.** *Plos One* 2010, **5**.

39. Mariappan V, Vellasamy KM, Thimma JS, Hashim OH, Vadivelu J: **Identification of immunogenic proteins from *Burkholderia cepacia* secretome using proteomic analysis.** *Vaccine* 2010, **28:**1318-1324.

40. Gupta MK, Subramanian V, Yadav JS: **Immunoproteomic identification of secretory and subcellular protein antigens and functional evaluation of the secretome fraction of *Mycobacterium immunogenum*, a newly recognized species of the *Mycobacterium chelonae*-*Mycobacterium abscessus* group.** *J Proteome Res* 2009, **8:**2319-2330.

41. Beltran PK, Gutierrez-Ortega A, Puebla-Perez AM, Gutierrez-Pabello JA, Flores-Valdez MA, Hernandez-Gutierrez R, Martinez-Velazquez M, Alvarez AH: **Identification of immunodominant antigens of *Mycobacterium bovis* by expression library immunization.** *Vet J* 2011, **190:**181-183.

42. Liao YH, Deng JH, Zhang AD, Zhou MG, Hu Y, Chen HC, Jin ML: **Immunoproteomic analysis of outer membrane proteins and extracellular proteins of *Actinobacillus pleuropneumoniae* JL03 serotype 3.** *BMC Microbiol* 2009, **9**.

43. Bunk S, Susnea I, Rupp J, Summersgill JT, Maass M, Stegmann W, Schrattenholz A, Wendel A, Przybylski M, Hermann C: **Immunoproteomic identification and serological responses to novel *Chlamydia pneumoniae* antigens that are associated with persistent *C. pneumoniae* infections.** *J Immunol* 2008, **180:**5490-5498.

44. Paliwal PK, Bansal A, Sagi SS, Sairam M: **Intraperitoneal immunization of recombinant HSP70 (DnaK) of *Salmonella typhi* induces a predominant Th2 response and protective immunity in mice against lethal *Salmonella* infection.** *Vaccine* 2011, **29:**6532-6539.

45. Delpino MV, Estein SM, Fossati CA, Baldi PC, Cassataro J: **Vaccination with *Brucella* recombinant DnaK and SurA proteins induces protection against *Brucella abortus* infection in BALB/c mice.** *Vaccine* 2007, **25:**6721-6729.

46. TeixeiraGomes AP, Cloeckaert A, Bezard G, Bowden RA, Dubray G, Zygmunt MS: **Identification and characterization of *Brucella ovis* immunogenic proteins using two-dimensional electrophoresis and immunoblotting.** *Electrophoresis* 1997, **18:**1491-1497.

47. Hu YH, Dang W, Deng T, Sun L: ***Edwardsiella tarda* DnaK: expression, activity, and the basis for the construction of a bivalent live vaccine against *E. tarda* and *Streptococcus iniae*.** *Fish Shellfish Immun* 2012, **32:**616-620.

48. Ling E, Feldman G, Portnoi M, Dagan R, Overweg K, Mulholland F, Chalifa-Caspi V, Wells J, Mizrachi-Nebenzahl Y: **Glycolytic enzymes associated with the cell surface of *Streptococcus pneumoniae* are antigenic in humans and elicit protective immune responses in the mouse.** *Clin Exp Immunol* 2004, **138:**290-298.

49. Sinha K, Bhatnagar R: **GroEL provides protection against *Bacillus anthracis* infection in BALB/c mice.** *Mol Immunol* 2010, **48:**264-271.

50. Plant KP, LaPatra SE, Cain KD: **Vaccination of rainbow trout, *Oncorhynchus mykiss* (Walbaum), with recombinant and DNA vaccines produced to *Flavobacterium psychrophilum* heat shock proteins 60 and 70.** *J Fish Dis* 2009, **32:**521-534.

51. Barker EN, Helps CR, Heesom KJ, Arthur CJ, Peters IR, Hofmann-Lehmann R, Tasker S: **Detection of humoral response using a recombinant heat shock protein 70, DnaK, of *Mycoplasma haemofelis* in experimentally and naturally *Hemoplasma*-infected cats.** *Clin Vaccine Immunol* 2010, **17:**1926-1932.

52. Lu C, Peng B, Li Z, Lei L, Li Z, Chen L, He Q, Zhong G, Wu Y: **Induction of protective immunity against *Chlamydia* *muridarum* intravaginal infection with the chlamydial immunodominant antigen macrophage infectivity potentiator.** *Microbes Infect* 2013.

53. Hung MC, Salim O, Williams JN, Heckels JE, Christodoulides M: **The *Neisseria meningitidis* macrophage infectivity potentiator protein induces cross-strain serum bactericidal activity and is a potential serogroup B vaccine candidate.** *Infect Immun* 2011, **79:**3784-3791.

54. Nowalk AJ, Gilmore RD, Carroll JA: **Serologic proteome analysis of *Borrelia burgdorferi* membrane-associated proteins.** *Infect Immun* 2006, **74:**3864-3873.

55. Shelburne CE, Shelburne PS, Dhople VM, Sweier DG, Giannobile WV, Kinney JS, Coulter WA, Mullally BH, Lopatin DE: **Serum antibodies to *Porphyromonas gingivalis* chaperone HtpG predict health in periodontitis susceptible patients.** *Plos One* 2008, **3**.

56. Zhang MJ, Meng FL, Cao FF, Qiao B, Liu GD, Liu HY, Zhou YZ, Dong HY, Gu YX, Xiao X et al.: **Cloning, expression, and antigenicity of 14 proteins from *Campylobacter jejuni*.** *Foodborne Pathog Dis* 2012, **9:**706-712.

57. O'Riordan AA, Morales VA, Mulligan L, Faheem N, Windle HJ, Kelleher DP: **Alkyl hydroperoxide reductase: a candidate *Helicobacter pylori* vaccine.** *Vaccine* 2012, **30:**3876-3884.

58. Tefon BE, Maass S, Ozcengiz E, Becher D, Hecker M, Ozcengiz G: **A comprehensive analysis of *Bordetella pertussis* surface proteome and identification of new immunogenic proteins.** *Vaccine* 2011, **29:**3583-3595.

59. Padmalayam I, Anderson B, Kron M, Kelly T, Baumstark B: **The 75-kilodalton antigen of *Bartonella bacilliformis* is a structural homolog of the cell division protein FtsZ.** *J Bact* 1997, **179:**4545-4552.

60. Pornwiroon W, Bourchookarn A, Paddock CD, Macaluso KR: **Proteomic analysis of *Rickettsia parkeri* strain Portsmouth.** *Infect Immun* 2009, **77:**5262-5271.

61. LaFrentz BR, Shoemaker CA, Klesius PH: **Immunoproteomic analysis of the antibody response obtained in Nile tilapia following vaccination with a *Streptococcus iniae* vaccine.** *Vet Microbiol* 2011, **152:**346-352.

62. Jain S, Afley P, Kumar S: **Immunological responses to recombinant cysteine synthase A of *Brucella abortus* in BALB/c mice.** *World J Microbiol Biotechnol* 2012.

63. Walz A, Mujer CV, Connolly JP, Alefantis T, Chafin R, Dake C, Whittington J, Kumar SP, Khan AS, DelVecchio VG: ***Bacillus anthracis* secretome time course under host-simulated conditions and identification of immunogenic proteins.** *Proteome Sci* 2007, **5:**11.

64. Zhou LY, Wang XH, Liu Q, Wang QY, Zhao Y, Zhang YX: **A novel multivalent vaccine based on secretary antigen-delivery induces protective immunity against *Vibrio anguillarum* and *Aeromonas hydrophila*.** *J Biotechnol* 2010, **146:**25-30.

65. Liang SQ, Wu HZ, Liu B, Xiao JF, Wang QY, Zhang YX: **Immune response of turbot (*Scophthalmus maximus* L.) to a broad spectrum vaccine candidate, recombinant glyceraldehyde-3-phosphate dehydrogenase of *Edwardsiella tarda*.** *Vet Immunol Immunopathol* 2012, **150:**198-205.

66. Kulkarni RR, Parreira VR, Sharif S, Prescott JF: **Immunization of broiler chickens against *Clostridium perfringens*-induced necrotic enteritis.** *Clin Vaccine Immunol* 2007, **14:**1070-1077.

67. Kulkarni RR, Parreira VR, Sharif S, Prescott JF: **Oral immunization of broiler chickens against necrotic enteritis with an attenuated *Salmonella* vaccine vector expressing *Clostridium perfringens* antigens.** *Vaccine* 2008, **26:**4194-4203.

68. Moore MM, Fernandez DL, Thune RL: **Cloning and characterization of *Edwardsiella ictaluri* proteins expressed and recognized by the channel catfish *Ictalurus punctatus* immune response during infection.** *Dis Aq Org* 2002, **52:**93-107.

69. Lowry JE, Isaak DD, Leonhardt JA, Vernati G, Pate JC, Andrews GP: **Vaccination with *Brucella abortus* recombinant in vivo-induced antigens reduces bacterial load and promotes clearance in a mouse model for infection.** *Plos One* 2011, **6**.

70. Hughes MJG, Moore JC, Lane JD, Wilson R, Pribul PK, Younes ZN, Dobson RJ, Everest P, Reason AJ, Redfern JM et al.: **Identification of major outer surface proteins of *Streptococcus agalactiae*.** *Infect Immun* 2002, **70:**1254-1259.

71. Hussain I, Mackie C, Cox D, Alderson R, Birkbeck TH: **Suppression of the humoral immune response of Atlantic salmon, *Salmo salar* L. by the 64 kDa serine protease of *Aeromonas salmonicida*.** *Fish Shellfish Immun* 2000, **10:**359-373.

72. Lund V, Espelid S, Mikkelsen H: **Vaccine efficacy in spotted wolffish *Anarhichas minor*: relationship to molecular variation in A-layer protein of atypical *Aeromonas salmonicida*.** *Dis Aq Org* 2003, **56:**31-42.

73. Lund V, Arnesen JA, Coucheron D, Modalsli K, Syvertsen C: **The *Aeromonas salmonicida* A-layer protein is an important protective antigen in oil-adjuvanted vaccines.** *Fish Shellfish Immun* 2003, **15:**367-372.

74. Sinyakov MS, Dror M, Zhevelev HM, Margel S, Avtalion RR: **Natural antibodies and their significance in active immunization and protection against a defined pathogen in fish.** *Vaccine* 2002, **20:**3668-3674.

75. Maurice S, Nussinovitch A, Jaffe N, Shoseyov O, Gertler A: **Oral immunization of *Carassius auratus* with modified recombinant A-layer proteins entrapped in alginate beads.** *Vaccine* 2004, **23:**450-459.

76. Arnesen KR, Mikkelsen H, Schroder MB, Lund V: **Impact of reattaching various *Aeromonas salmonicida* A-layer proteins on vaccine efficacy in Atlantic cod (*Gadus morhua*).** *Vaccine* 2010, **28:**4703-4708.

77. Anuradha K, Foo HL, Mariana NS, Loh TC, Yusoff K, Hassan MD, Sasan H, Raha AR: **Live recombinant *Lactococcus lactis* vaccine expressing aerolysin genes D1 and D4 for protection against *Aeromonas hydrophila* in tilapia (*Oreochromis niloticus*).** *J Appl Microbiol* 2010, **109:**1632-1642.

78. Ni XD, Wang N, Liu YJ, Lu CP: **Immunoproteomics of extracellular proteins of the *Aeromonas hydrophila* China vaccine strain J-1 reveal a highly immunoreactive outer membrane protein.** *FEMS Immunol Med Mic* 2010, **58:**363-373.

79. Zhang C, Yu Z, Tian L, Zhao B: **Cloning and expression of a hemolysin gene of *Aeromonas hydrophila* and the immunogenicity of the toxoid.** *Chinese J Biotechnol* 2009, **25:**251-256.

80. Vigil PD, Alteri CJ, Mobley HLT: **Identification of in vivo-induced antigens including an RTX family exoprotein required for uropathogenic *Escherichia coli* virulence.** *Infect Immun* 2011, **79:**2335-2344.

81. Paton AW, Paton JC: **Reactivity of convalescent-phase hemolytic-uremic syndrome patient sera with the megaplasmid-encoded TagA protein of *Shiga* toxigenic *Escherichia coli* O157.** *J Clin Microbiol* 2002, **40:**1395-1399.

82. Wu L, Jiang YN, Tang Q, Lin HX, Lu CP, Yao HC: **Development of an *Aeromonas hydrophila* recombinant extracellular protease vaccine.** *Microb Pathogenesis* 2012, **53:**183-188.

83. Kudva IT, Griffin RW, Garren JA, Calderwood SB, John M: **Identification of a protein subset of the anthrax spore immunome in humans immunized with the anthrax vaccine adsorbed preparation.** *Infect Immun* 2005, **73:**5685-5696.

84. Cao J, Chen TM, Li DR, Wong CK, Chen DP, Xu WC, Zhang XM, Lam CWK, Yin YB: **Mucosal immunization with purified ClpP could elicit protective efficacy against pneumococcal pneumonia and sepsis in mice.** *Microbes Infect* 2008, **10:**1536-1542.

85. Cao J, Chen DP, Xu WC, Chen TM, Xu SX, Luo JY, Zhao Q, Liu BZ, Wang DS, Zhang XM et al.: **Enhanced protection against pneumococcal infection elicited by immunization with the combination of PspA, PspC, and ClpP.** *Vaccine* 2007, **25:**4996-5005.

86. Mayfield JE, Bricker BJ, Godfrey H, Crosby RM, Knight DJ, Halling SM, Balinsky D, Tabatabai LB: **The cloning, expression, and nucleotide-sequence of a gene coding for an immunogenic *Brucella abortus* protein.** *Gene* 1988, **63:**1-9.

87. Sakai T, Matsuyama T, Nishioka T, Nakayasu C, Kamaishi T, Yamaguchi K, Iida T: **Identification of major antigenic proteins of *Edwardsiella tarda* recognized by Japanese flounder antibody.** *J Vet Diag Invest* 2009, **21:**504-509.

88. Crump EM, Burian J, Allen PD, Gale S, Kay WW: **Identification of a ribosomal L10-like protein from *Flavobacterium psychrophilum* as a recombinant vaccine candidate for rainbow trout fry syndrome.** *J Mol Microbiol Biotech* 2007, **13:**55-64.

89. Shim DH, Chang SY, Park SM, Jang H, Carbis R, Czerkinsky C, Uematsu S, Kweona MN, Kweon MN: **Immunogenicity and protective efficacy offered by a ribosomal-based vaccine from *Shigella flexneri* 2a.** *Vaccine* 2007, **25:**4828-4836.

90. Johnson W: **Ribosomal vaccines .II. Specificity of immune response to ribosomal ribonucleic-acid and protein isolated from *Salmonella typhimurium*.** *Infect Immun* 1973, **8:**395-400.

91. Normier G, Pinel AM, Dussourd dL, Wigzell H, Binz H: **Ribosomes as carriers for antigenic determinants of the surface of micro-organisms.** *Dev Biol Stand* 1992, **77:**79-85.

92. Goodin JL, Raab RW, Mckown RL, Coffman GL, Powell BS, Enama JT, Ligon JA, Andrews GP: ***Yersinia pestis* outer membrane type III secretion protein YscC: expression, purification, characterization, and induction of specific antiserum.** *Protein Expres Purif* 2005, **40:**152-163.

93. Bansal A, Paliwal PK, Sagi SSK, Sairam M: **Effect of adjuvants on immune response and protective immunity elicited by recombinant Hsp60 (GroEL) of *Salmonella typhi* against *S. typhi* infection.** *Mol Cell Biochem* 2010, **337:**213-221.

94. Paliwal PK, Bansal A, Sagi SS, Mustoori S, Govindaswamy I: **Cloning, expression and characterization of heat shock protein 60 (groEL) of *Salmonella enterica* serovar *typhi* and its role in protective immunity against lethal *Salmonella* infection in mice.** *Clin Immunol* 2008, **126:**89-96.

95. Yamaguchi H, Osaki T, Taguchi H, Sato N, Toyoda A, Takahashi M, Kai M, Nakata N, Komatsu A, Atomi Y et al.: **Effect of bacterial flora on postimmunization gastritis following oral vaccination of mice with *Helicobacter pylori* heat shock protein 60.** *Clin Diag Lab Immunol* 2003, **10:**808-812.

96. Thomas S, Thirumalapura NR, Crocquet-Valdes PA, Luxon BA, Walker DH: **Structure-based vaccines provide protection in a mouse model of ehrlichiosis.** *Plos One* 2011, **6**.

97. Bae JE, Schurig GG, Toth TE: **Mice immune responses to *Brucella abortus* heat shock proteins - Use of baculovirus recombinant-expressing whole insect cells, purified *Brucella abortus* recombinant proteins, and a vaccinia virus recombinant as immunogens.** *Vet Microbiol* 2002, **88:**189-202.

98. Choi J, Lee SY, Kim K, Choi BK: **Identification of immunoreactive epitopes of the *Porphyromonas gingivalis* heat shock protein in periodontitis and atherosclerosis.** *J Periodontal Res* 2011, **46:**240-245.

99. Lutwyche P, Exner MM, Hancock REW, Trust TJ: **A conserved *Aeromonas salmonicida* porin provides protective immunity to rainbow-trout.** *Infect Immun* 1995, **63:**3137-3142.

100. Gong Q, Qu N, Niu M, Qin C, Cheng M, Sun X, Zhang A: **Immune responses and protective efficacy of a novel DNA vaccine encoding outer membrane protein of avian *Pasteurella multocida*.** *Vet Immunol Immunopathol* 2013.

101. Erova TE, Rosenzweig JA, Sha J, Suarez G, Sierra JC, Kirtley ML, van Lier CJ, Telepnev MV, Motin VL, Chopra AK: **Evaluation of protective potential of *Yersinia pestis* outer membrane protein antigens as possible candidates for a new-generation recombinant plague vaccine.** *Clin Vaccine Immunol* 2013, **20:**227-238.

102. Ayalew S, Shrestha B, Montelongo M, Wilson AE, Confer AW: **Immunogenicity of *Mannheimia haemolytica* recombinant outer membrane proteins serotype 1-specific antigen, OmpA, OmpP2, and OmpD15.** *Clin Vaccine Immunol* 2011, **18:**2067-2074.

103. Fang HM, Ge RW, Sin YM: **Cloning, characterisation and expression of *Aeromonas hydrophila* major adhesin.** *Fish Shellfish Immun* 2004, **16:**645-658.

104. Merino S, Vilches S, Canals R, Ramirez S, Tomas JM: **A C1q-binding 40 kDa porin from *Aeromonas salmonicida*: Cloning, sequencing, role in serum susceptibility and fish immunoprotection.** *Microb Pathog* 2005, **38:**227-237.

105. Wang N, Yang Z, Zang MF, Liu YJ, Lu CP: **Identification of Omp38 by immunoproteomic analysis and evaluation as a potential vaccine antigen against *Aeromonas hydrophila* in Chinese breams.** *Fish Shellfish Immun* 2013, **34:**74-81.

106. Maiti B, Shetty M, Shekar M, Karunasagar I, Karunasagar I: **Evaluation of two outer membrane proteins, Aha1 and OmpW of *Aeromonas hydrophila* as vaccine candidate for common carp.** *Vet Immunol Immunopathol* 2012, **149:**298-301.

107. Guan RZ, Xiong J, Huang WS, Guo SL: **Enhancement of protective immunity in European eel (*Anguilla anguilla*) against *Aeromonas hydrophila* and *Aeromonas sobria* by a recombinant *Aeromonas* outer membrane protein.** *Acta Bioch et Bioph Sin* 2011, **43:**79-88.

108. Li NQ, Yang ZH, Bai JJ, Fu XZ, Liu LH, Shi CB, Wu SQ: **A shared antigen among *Vibrio* species: outer membrane protein-OmpK as a versatile Vibriosis vaccine candidate in Orange-spotted grouper (*Epinephelus coioides*).** *Fish Shellfish Immun* 2010, **28:**952-956.

109. Mao ZJ, Yu L, You ZQ, Wei YW, Liu Y: **Cloning, expression and immunogenicty analysis of five outer membrane proteins of *Vibrio parahaemolyticus* zj2003.** *Fish Shellfish Immun* 2007, **23:**567-575.

110. Bi YJ, Du ZM, Han YP, Guo ZB, Tan YF, Zhu ZW, Yang RF: ***Yersinia pestis* and host macrophages: immunodeficiency of mouse macrophages induced by YscW.** *Immunology* 2009, **128:**e406-e417.

111. Karlsen C, Espelid S, Willassen NP, Paulsen SM: **Identification and cloning of immunogenic *Aliivibrio salmonicida* Pal-like protein present in profiled outer membrane and secreted subproteome.** *Dis Aq Org* 2011, **93:**215-223.

112. Paul-Satyaseela M, Karched M, Bian Z, Ihalin R, Boren T, Arnqvist A, Chen C, Asikainen S: **Immunoproteomics of *Actinobacillus actinomycetemcomitans* outer-membrane proteins reveal a highly immunoreactive peptidoglycan-associated lipoprotein.** *J Med Microbiol* 2006, **55:**931-942.

113. Carbonetti NH, Artamonova GV, Andreasen C, Dudley E, Mays RM, Worthington ZEV: **Suppression of serum antibody responses by pertussis toxin after respiratory tract colonization by *Bordetella pertussis* and identification of an immunodominant lipoprotein.** *Infect Immun* 2004, **72:**3350-3358.

114. Burnens A, Stucki U, Nicolet J, Frey J: **Identification and characterization of an immunogenic outer-membrane protein of *Campylobacter jejuni*.** *J Clin Microbiol* 1995, **33:**2826-2832.

115. Yoon WS, Park SH, Park YK, Park SC, Sin JI, Kim MJ: **Comparison of responses elicited by immunization with a *Legionella* species common lipoprotein delivered as naked DNA or recombinant protein.** *DNA Cell Biol* 2002, **21:**99-107.

116. Khushiramani RM, Maiti B, Shekar M, Girisha SK, Akash N, Deepanjali A, Karunasagar I, Karunasagar I: **Recombinant *Aeromonas hydrophila* outer membrane protein 48 (Omp48) induces a protective immune response against *Aeromonas hydrophila* and *Edwardsiella tarda*.** *Res Microbiol* 2012, **163:**286-291.

117. Yang TC, Ma XC, Liu F, Lin LR, Liu LL, Liu GL, Tong ML, Fu ZG, Zhou L: **Screening of the *Salmonella paratyphi* A CMCC 50973 strain outer membrane proteins for the identification of potential vaccine targets.** *Mol Med Rep* 2012, **5:**78-83.
